# Supplementary material for: In vivo three-dimensional multispectral photoacoustic imaging of dual enzyme-driven cyclic cascade reaction for tumor catalytic therapy
Source: Nat Commun. 2022 Mar 11;13:1298. doi: 10.1038/s41467-022-29082-1 (PMC8917194; doi:10.1038/s41467-022-29082-1)
Supplement: Supplementary file 1 — Supplementary Information [file 41467_2022_29082_MOESM1_ESM.pdf]

Supplementary Information

In vivo three-dimensional multispectral photoacoustic imaging of dual enzyme-driven cyclic cascade reaction for tumor catalytic therapy

*Shan Lei[1,2,3], Jing Zhang[1,3], Nicholas Thomas Blum[1], Meng Li[1], Dong-Yang Zhang[1], Weimin Yin[1], Feng zhao[1], Jing Lin[1], and Peng Huang[1]**

[1]Marshall Laboratory of Biomedical Engineering, International Cancer Center, Laboratory of Evolutionary Theranostics (LET), School of Biomedical Engineering, Shenzhen University Health Science Center, Shenzhen 518060, China

[2]Key Laboratory of Optoelectronic Devices and Systems of Ministry of Education and Guangdong Province, College of Physics and Optoelectronic Engineering, Shenzhen University, Shenzhen 518060, China

[3]These authors contributed equally: Shan Lei, Jing Zhang

(Email: peng.huang@szu.edu.cn)

Supplementary methods

Materials. Palladium(II) acetylacetonate ($Pd(acac)_2$), hexacarbonylmolybdenum ($Mo(CO)_6$), oleylamine, H_2O_2 (30%), 3,3',5,5'-tetramethylbenzidine (TMB), 1 methacrylic acid (MAA), pentaerythritol tetrakis 3-mercaptopropionate (PTMP), ethanol, azodiisobutyronitrile (AIBN), Triton X-100, paraformaldehyde and were purchased from Sigma-Aldrich Co., Ltd. 3-(4,5-Dimethylthiazol-2-yl)-2,5-diphenyltetrazolium bromide (MTT), calcein AM, propidium iodide (PI), reactive oxygen species (ROS) assay kit (2',7'-dichlorofluorescin diacetate, DCFH-DA), 4',6'-diamidino-2-phenylidole (DAPI), hydroxyphenyl fluorescein (HPF), Annexin V-FITC/PI Apoptosis Assay Kit and penicillin-streptomycin solution (100X) were purchased from Beyotime Institute of Biotechnology (Shanghai, China). Glucose oxidase (GOx, 100000-250000 units g^{-1}) was purchased from Sigma-Aldrich. C11 BODIPY 581/591 was purchased from Cayman Chemical. Fetal bovine serum (FBS) and Dulbecco's modified Eagle's medium (DMEM) were purchased from GIBCO Co., Ltd. 4T1 cells were purchased from Cell Bank of Chinese Academy of Sciences (Shanghai, China). The thioether polymer ligands (PTMP-PMAA) were prepared according to the previous study[1].

Characterization. The morphologies of the prepared nanosheets were observed by transmission electron microscopy (TEM, Hitachi H-7700, 100 kV). High-resolution transmission electron microscopy (HRTEM) and high-angle annular dark field-scanning transmission electron microscopy (HAADF-STEM) were carried out on a FEI Tecnai F200 microscope equipped with an Oxford energy-dispersive X-ray (EDX) analysis system. The STEM images were processed by Digital Micrograph Gatan software packages with GPA script to obtain the strain mapping[2, 3]. The FT-IR spectra of the samples in KBr plate were measured on FT-IR spectrometer (Spectrum Two, PerkinElmer, USA). X-ray photoelectron spectroscopy (XPS) was carried out with Thermo ESCALAB 250 using monochromatic Al Kα radiation. The UV-Vis-NIR spectra of the aqueous dispersions of nanoparticles were measured with Lambda 1050+ spectrometer (PerkinElmer) and analyzed by UV Winlab (Version 7.1). Zeta potential was measured on Zetasizer Nano-ZS90 (Malven, England). Cell experiments were conducted on High Content System (PerkinElmer).

Calculation of photothermal conversion efficiency. The photothermal conversion efficiency (η) of PMNS was calculated according to the previously reported methods[4], detailed calculation as following:

During the photothermal heating process, the total energy balance for the system can be expressed as:

Supplementary Equation (1): $\sum_i m_i C_{P,i} \frac{d\Delta T}{dt} = Q_{NP} + Q_S - Q_{Loss}$

Where m (g) represents the mass of the solution (m_s) and sample cuvette (m_q), C (J g^{-1} ^{o}C^{-1}) includes the constant-pressure heat capacity of solution (c_s) and sample cuvette (c_q), ΔT (°C) is the difference between the solution temperature T at time t and the starting solution temperature T_0, Q_{NP} (mW) is determined as the energy arising from the nanoparticles, and Q_{Loss} (mW) is the thermal energy lost to the surrounding environment. In addition, Q_S (mW) is the energy input by the sample cuvette and the solvent (pure DI water).

For Q_{NP}, Supplementary Equation (2) can be given as:

Supplementary Equation (2): $Q_{NP} = I\,(1 - 10^{-A_{1064}})\eta$

Where I is the laser power which is incident on the system, A_{1064} is defined as the absorbance of the PMNS at the wavelength of 1064 nm, and η is known as the photothermal conversion efficiency from the absorbed laser energy to thermal energy.

Furthermore, the energy dissipation mainly occurs through the heat conduction and thermal radiation. Q_{Loss} is linear with temperature for the outgoing thermal energy, then take the form as Supplementary Equation (3):

Supplementary Equation (3): $Q_{Loss} = \hbar S\Delta T = \hbar S(T - T_{Sur})$

Where h (mW m^{-2}·^{o}C^{-1}) is heat transfer coefficient, S (m^2) is the surface area of the container, ΔT is the temperature change which is defined as T- T_{sur}, T (^{o}C) is the water temperature and T_{Sur} (^{o}C) is the solution temperature ambient temperature of surrounding environment.

When the temperature rises at a maximum steady-state temperature T_{Max} (^{o}C), the system reaches the steady state. In this case, the heat input is equal to heat output, and the left side of Supplementary Equation (1) becomes zero. We then obtain

Supplementary Equation (4): $Q_{NP} + Q_S = Q_{Loss} = \hbar S\,(T_{Max} - T_{Sur})$

Then η can be determined by combining Supplementary Equation (1-4) and rearranging:

Supplementary Equation (5): $\eta = [\hbar S(T_{Max} - T_{Sur}) - Q_s] \,/\, I(1 - 10^{-A_{808}})$

Where Q_S is measured independently, the (T_{Max}-T_{Sur}) is 48.70 ^oC, I is 600 mW, A_{1064} is 1.509 (Figure S4). Thus, in the Supplementary Equation (5), only the hS remains unknown parameter for calculating η.

In order to solve hS, the following notation θ is used herein, which is defined as the ratio of (T-T_{sur}) to (T_{Max}-T_{sur}):

Supplementary Equation (6): $\theta = (T - T_{Sur})/(T_{Max} - T_{Sur})$

And a sample system time constant τ_S (s) is introduced:

Supplementary Equation (7): $\tau_s = \frac{\sum_i m_i c_{P,i}}{hs}$

Substituting Supplementary Equations (6) and (7) into Supplementary Equation (1) and rearranging to obtain:

Supplementary Equation (8): $\frac{d\theta}{d\tau} = \frac{1}{\tau_s}\left[\frac{Q_{NF}+Q_S}{hS(T_{max}-T_{Sur})} - \theta\right]$

When at the cooling stage of PMNS aqueous dispersion, the laser source has been shut off, so the Q_{NP} + Q_S= 0. Under this condition, Supplementary Equation (8) becomes:

Supplementary Equation (9): $dt = -\tau_s\,(d\theta/\theta)$

Note that after integration Supplementary Equation (9), the Supplementary Equation expresses as:

Supplementary Equation (10): $t = -\tau_s \ln\theta$

Therefore, time constant for heat transfer from the system is determined to be τ_S = 145.59 s by applying the linear time data from the cooling period *vs* –lnθ (Figure 3e). In addition, the m is 0.25 g and the C is 4.2 J g^{-1} $^oC^{-1}$. Thus, according to Supplementary Equation (7), the hS is calculated to be 7.21 mW $^oC^{-1}$. Substituting hS = 7.21 mW $^oC^{-1}$ into Supplementary Equation (5), the result photothermal conversion efficiency (η) of PMNS at 1064 nm can be calculated to be 60.4%.

The result of that case irradiated under 808 nm laser is that τ_s = 170.98 s, obtained by linear fitting using linear cooling time and negative natural logarithm of temperature (Figure 3f). The value of m and C are 0.25 g and 4.2 J g^{-1} $^oC^{-1}$ respectively. Therefore, hS is calculated to be 6.14 mW $^oC^{-1}$ by using Supplementary Equation (7). In addition, the (T_{Max}-T_{Sur}) is 51.13 ^oC, I_{808} is 600 mW, A_{808} is 1.691. Thus, the photothermal conversion efficiency (η) of PMNS at 808 nm can be calculated to be 51.3%.

Cell uptake assay. For the cytotoxicity evaluation, 4T1 cells were cultured in a 96-well plate at a density of 1 × 10^4 cells/well at 37 ^{o}C with 5% CO_2 humidified atmosphere for 24 h. Subsequently, the culture medium was removed. The cells were incubated with fresh medium containing IR680 labeled PMNSG, the real-time fluorescence signals were measured by High Content System (PerkinElmer).

Apoptosis and necrosis assay. 4T1 cancer cells were seeded in 6-well plates under hypoxic condition at 37 ^{o}C for 24 h. Subsequently, the medium was removed and incubated with fresh medium containing PBS, GOx, PMNS and PMNSG. for 4 h, the cells were irradiated with 1064-nm irradiation (0.4 W cm^{-2}, 5 min). After that, the cells were co-cultured annexin V-FITC/PI apoptosis detection kit for another 4 h. Finally, all treated cells were harvested and quantified by using flow cytometer and analyzed by FlowJo_V10 (version 10.7.1).

In vivo toxicity examination. For the long-term biocompatibility study, mice were divided into two groups (n = 3) randomly with different treatment. After 14 days of monitoring, about 0.8 mL of blood per mouse from orbital venous plexus collected was employed for complete blood biochemistry assay, and the major organs (heart, spleen, kidney, liver, and lung) were collected after euthanasia of the mice. These organs were fixed in 4% paraformaldehyde and stained with hematoxylin and eosin (H & E) for histological analysis.

Pharmacokinetics, Biodistribution, and Metabolism Studies. For the pharmacokinetic experiments, 4T1-tumor-bearing mice were intravenously injected with PMNSG (10 mg kg^{-1}) and intraperitoneally injected with 5% glucose solution (200 µL) in PBS (n = 3). About 80 µL aliquot of blood was collected from the orbital vein plexus at varied time intervals (0, 5, and 10 min, 0.5, 1, 2, 4, 8, 24 and 48 h). Quantitative analysis of Pd element was measured by ICP-OES. The double component pharmacokinetic model was used to determine the terminal half-life of PMNSG in vivo blood. And the biodistribution of PMNSG in tumor and other tissues were also evaluated, after intravenous injection of PMNSG (10 mg kg^{-1}), at each prefixed time points (0, 4, 12, 24, and 48 h), one group of 4T1-tumor-bearing mice (n = 3) were euthanized to obtain the tumors and other main organs. Followed by weighed, homogenized, and treated with strong acid, the PMNSG distributions in different tissues were measured by ICP-OES and calculated as the percentage of injected dose per gram of tissue. To

quantitatively detect the metabolism process, PMNSG (10 mg kg^{-1}) in PBS were intravenously injected into the 4T1-tumor-bearing mice (n = 6), then feces and urine of the mice were collected in the following five days. The Pd contents in urine and feces were quantitatively determined by ICP-OES.

PA analysis of hemoglobin oxygen saturation (sO_2). For monitoring the variation of oxygen content in tumor tissue during therapy, the PA-Mode 3D (Multiwavelength) and PA-Mode 3D (Oxy-Hemo) of Vevo LAZR-X system was employed to record corresponding signals. The parameters of the system were listed as follows:

Scanning time: single-spectral photoacoustic imaging (about 1.5 min) and multispectral photoacoustic imaging (about 8 min); scanning area: ~450 mm^2; pulse repetition rate: 20 Hz; safe energy: 20 mJ/cm^2.

Supplementary Table 1. The setting parameters of the PA system

PA-Mode 3D (Oxy-Hemo)	Parameters
Frequency	40 MHz
Wavelength Range	680-970 nm
PA Gain	40 dB
Gain	29 dB
Depth, Width	15.00, 14.08 mm
Wavelengths	680, 730, 780, 916, 924, 950
PA Acquisition	sO_2/HbT
3D Method	Alt
3D Step Size	0.20 mm
Display Type	OxyZated
Threshold HbT	20
sO_2 Range	0-100%

The calculation of hemoglobin oxygen saturation (sO_2) based on PA intensities of

hemoprotein can be simply defined as follows: sO_2 = Oxyhemoglobin PA intensity/(Dexyhemoglobin PA intensity + Oxyhemoglobin PA intensity), above that, (Deoxyhemoglobin PA intensity + Oxyhemoglobin PA intensity) = Hemoglobin-Total (HbT). Before and post intravenously administered with PBS (100 uL, Cat. No. SH30256.01, HyClone), GOx (dose of 0.2 mg kg^{-1}), PMNS (dose of 10 mg kg^{-1}) and PMNSG (dose of 10 mg kg^{-1}), the corresponding signals of each group of mice (n = 3) was collected by the built-in module of LAZR system at prefixed time points (0, 1, 2, 4, 8 h). Due to the inevitable PA intensity spectrum overlap among PMNS, PMNSG, oxyhemoglobin and deoxyhemoglobin, the Oxy-Hemo mode with default setting of the system was inadequate to eliminate interference signals of PMNS or PMNSG, thus accurately reflecting the oxygen variation of tumor area. Therefore, the multiwavelength mode together with unmixing processing was employed. The PA spectrum of PMNS and PMNSG at same concentration (20 μg mL^{-1}) served as components for the following unmixing process were collected. Before and post intravenously administered with PBS (100 uL, Cat. No. SH30256.01, HyClone), GOx (dose of 0.2 mg kg^{-1}), PMNS (dose of 10 mg kg^{-1}) and PMNSG (dose of 10 mg kg^{-1}), the corresponding signals of each group of mice (n = 3) was collected and unmixed automatically by Vevo LAZR system.

Supplementary Table 2. The setting parameters of the PA system

Multiwavelength	Parameters
Frequency	40 MHz
Wavelength Range	680-970 nm
PA Gain	40 dB
Gain	29 dB
Depth/Width	15.00/14.08 mm
Components	PMNS, OxyHemo, DeoxyHemo
Wavelengths	680, 730, 780, 916, 924, 950
PA Acquisition	Unmixing
3D Method	Alt
3D Step Size	0.20 mm

Pearson correlation analysis among deoxyHb, oxyHb and PMNSG (or PMNS). Pearson product-moment correlation coefficient (PPMCC) is a measure of the degree of correlation between two variables. It is a value between 1 and -1, where 1 means the variable is completely positively correlated, 0 means irrelevant, and -1 means completely negatively correlated. The calculation formula is as follows:

Supplementary Equation (11): $r = \frac{\sum_{i=1}^{n}(x_i-\bar{x})(y_i-\bar{y})}{\sqrt{\sum_{i=1}^{n}(x_i-\bar{x})^2}\sqrt{\sum_{i=1}^{n}(y_i-\bar{y})^2}}$ (n=1,2,3,4,……)

Where ***r*** is the Pearson correlation coefficient, ***x*** and ***y*** are the two variables among separated PA signals of OxyHb, DeoxyHb and PMNSG (or PMNS).

Supplementary Table 3. The degree of correlation represented by the ***r*** value.

**|*r*|**	Degree of correlation
0.8-1.0	very strong
0.6-0.8	strong
0.4-0.6	general
0.2-0.4	weak
0.0-0.2	irrelevant

Supplementary Figures:

The transmission electron microscopy (TEM) image shows that the PMBNs is dominated by sheet-like morphology with an average diameter of 56 nm (Supplementary Fig. 1a). The lattice spacing of 0.23 nm obtained from high-resolution transmission electron microscopy (HRTEM) image (Supplementary Fig. 1b, inset) corresponded well to the plane of the Pd (111) facet[5, 6]. The elemental mapping reveal that the Pd and Mo were homogenously distributed on the nanosheets (Fig. 1a). The powder X-ray diffraction (XRD) pattern suggests that the PMBNs possess a face-centered cubic structure (Supplementary Fig. 3), in agreement with a previous report[7]. X-ray photoelectron spectroscopy (XPS) (Supplementary Fig. 4a) further proves the presence of Pd and Mo in PMBNs. The high resolution XPS spectra (Supplementary Fig. 4b and Fig. 4c) of Mo 3*d* and Pd 3*d* confirm that the Mo and Pd in bimetallene materials are mainly in their metallic state[7].

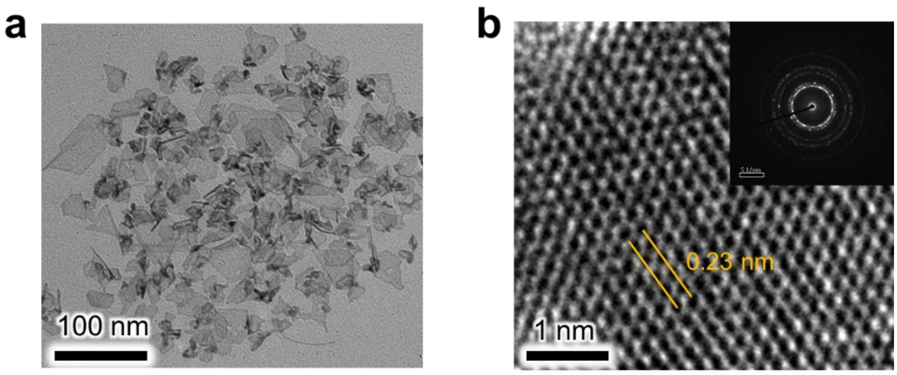

Supplementary Fig.1 (a) TEM image of PdMo bimetallene. **(b)** HRTEM image of PdMo bimetallene. Inset, the corresponding fast Fourier transform patterns.

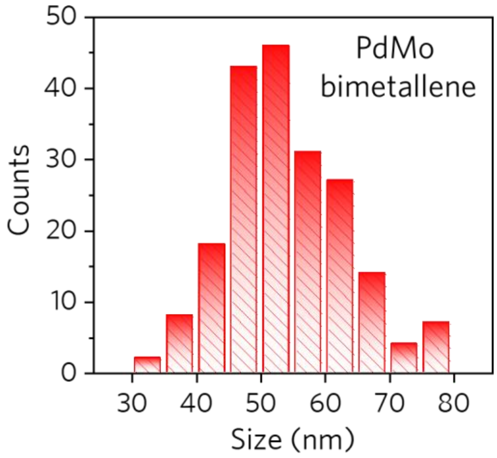

Supplementary Fig. 2 Size distribution of PdMo bimetallene nanosheets. Source data are provided as a Source Data file.

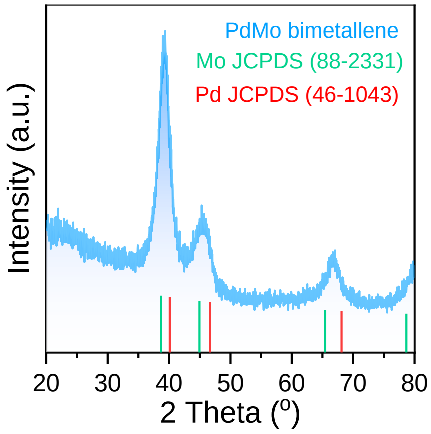

Supplementary Fig. 3 XRD spectra of PdMo bimetallene. Source data are provided as a Source Data file.

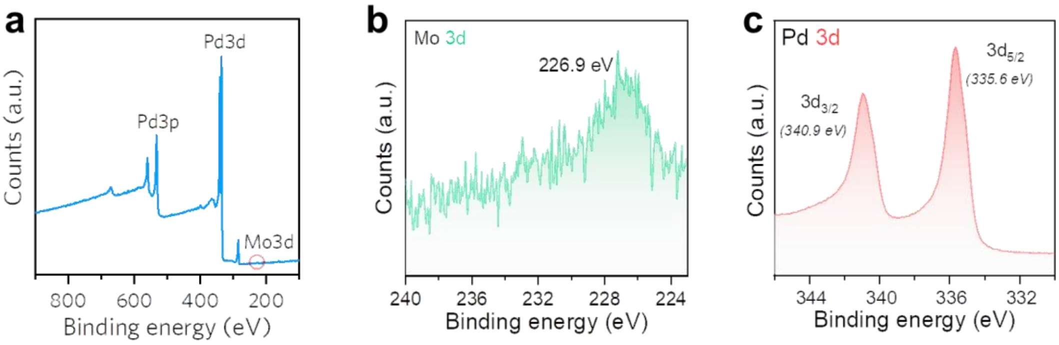

Supplementary Fig. 4 (a) XPS spectrum of PdMo bimetallene nanosheets. High resolution XPS spectrum of **(b)** Mo 3d and **(c)** Pd 3d for PdMo bimetallene nanosheets. Source data are provided as a Source Data file.

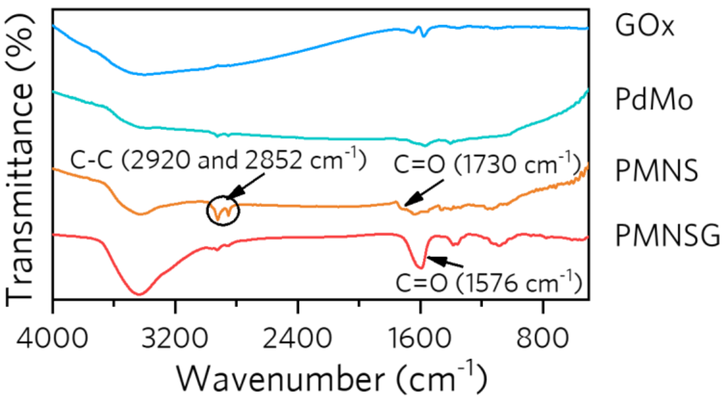

Supplementary Fig. 5 FT-IR spectra of GOx, PdMo bimetallene, PMNS and PMNSG. Source data are provided as a Source Data file.

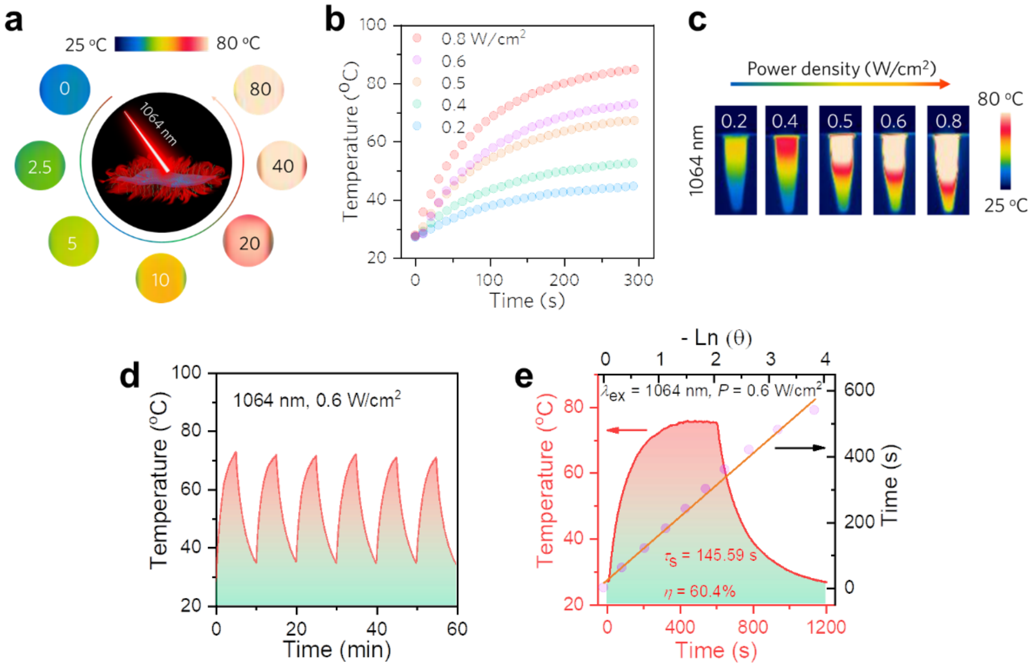

Supplementary Fig. 6 (a) IR thermal images of PMNS dispersion at different concentration under 1064-nm laser irradiation (0.6 W cm^{-2}) for 5 min. **(b)** Power density-dependent photothermal heating curves of PMNS under 1064-nm laser irradiation for 5 min and the corresponding IR thermal images **(c)**. **(d)** Photothermal stability (1064 nm, 0.6 W cm^{-2}). **(e)** Real-time temperature changes of PMNS dispersion (40 μg mL^{-1}) during 10-min's irradiation of 1064-nm (0.6 W cm^{-2}) laser and the following 10-min's natural cooling process, as well as the corresponding cooling time versus -lnθ derived from the data of the cooling period. Source data are provided as a Source Data file.

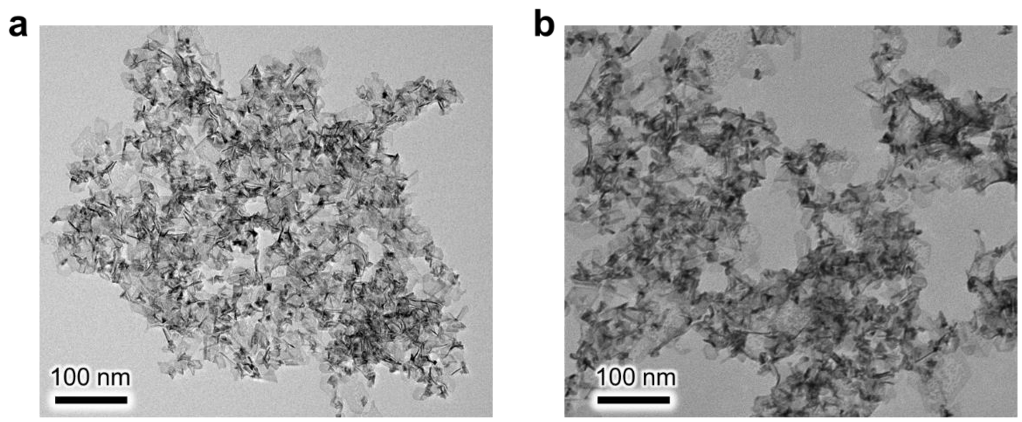

Supplementary Fig. 7 TEM images of PMNS before **(a)** and after 1064-nm laser irradiation for 20 min **(b).**

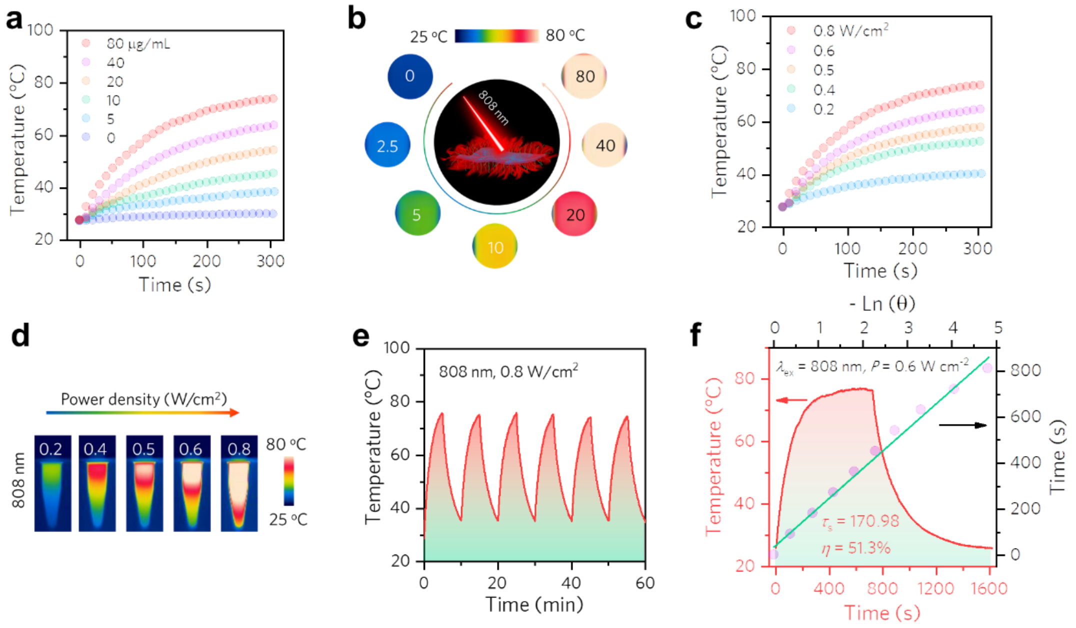

Supplementary Fig. 8 (a) Concentration-dependent photothermal heating curves of PMNS (0, 5, 10, 20, 40, 80 μg mL^{-1}) under 808-nm laser irradiation for 5 min and **(b)** its corresponding IR thermal images. **(c)** Power density-dependent photothermal heating curves of PMNS under 808-nm laser irradiation for 5 min and its **(d)** corresponding IR thermal images. **(e)** Photothermal stability (1064 nm, 0.6 W cm^{-2}). **(f)** Real-time temperature changes of PMNS dispersion (40 μg mL^{-1}) during 10-min's irradiation of 808-nm (0.6 W cm^{-2}) laser and the following 10-min's natural cooling process, as well as the corresponding cooling time versus -lnθ derived from the data of the cooling period. Source data are provided as a Source Data file.

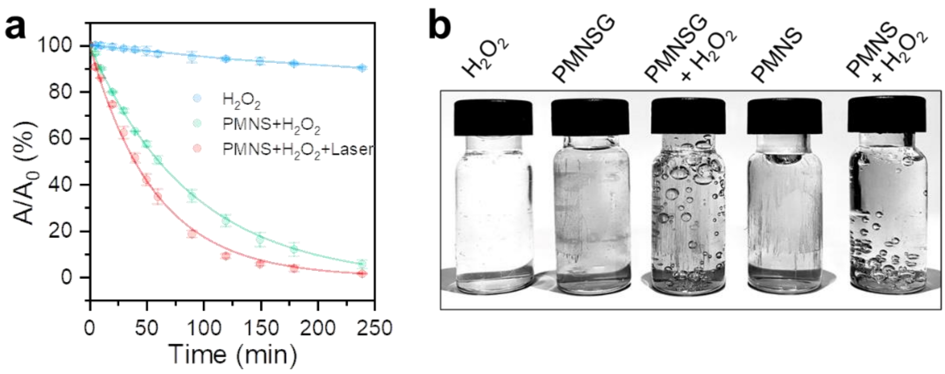

Supplementary Fig. 9 (a) H_2O_2 consumption in different treatments (H_2O_2, H_2O_2+PMNS and H_2O_2+PMNS+laser), respectively. Data are presented as mean ± SD. (n = 3). **(b)** Optical images of different PBS (pH = 7.4) solutions (from left to right: only H_2O_2; only PMNSG; PMNSG + H_2O_2; only PMNS; PMNS + H_2O_2) to prove the oxygen generation. Source data are provided as a Source Data file.

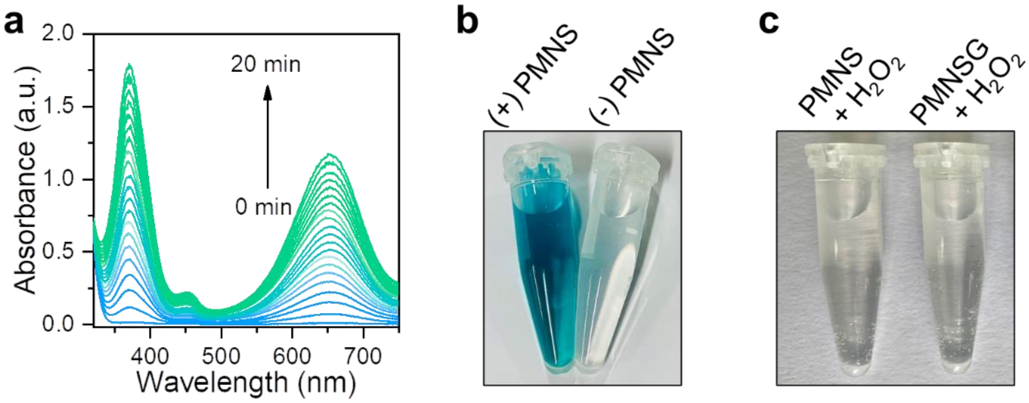

Supplementary Fig. 10 (a) Time-dependent TMB oxidation indicating the POD-like activity of PMNS**. (b)** Optical images of TMB solution (HAc-NaAc buffer solution: 0.1M, pH 4.5) with and without addition of PMNS. **(c)** Optical images of TMB PBS (pH = 7.4) solutions under different conditions. Source data are provided as a Source Data file.

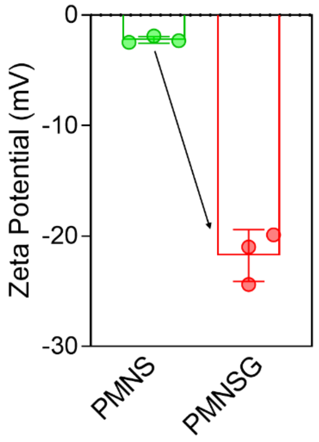

Supplementary Fig. 11 Zeta potential of PMNS and PMNSG. Data are presented as mean ± SD. (n = 3). Source data are provided as a Source Data file.

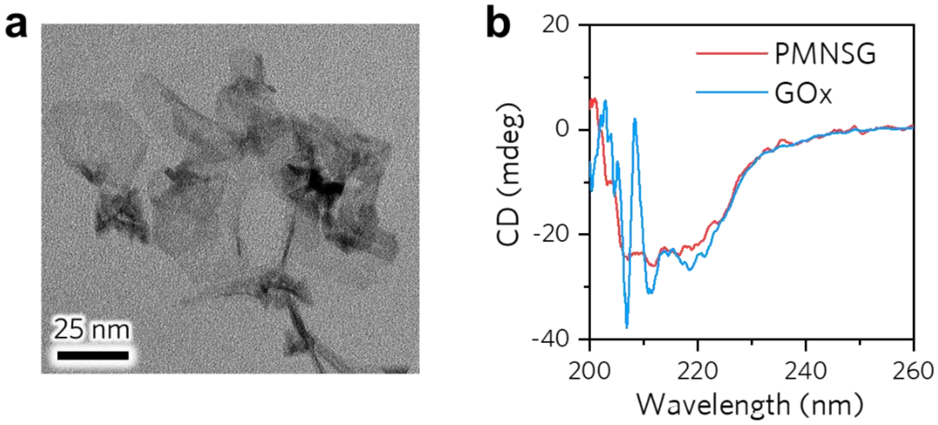

Supplementary Fig. 12 (a) TEM image of PMNSG. **(b)** Circular dichroism (CD) spectra of aqueous solutions of PMNSG and GOx. Source data are provided as a Source Data file.

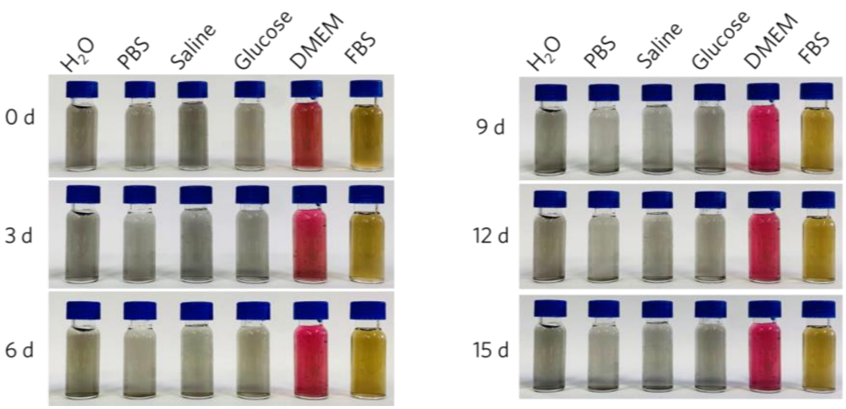

Supplementary Fig. 13 The dispersibility of PMNS in different physiological media (H_2O, PBS, saline, glucose, DMEM and FBS).

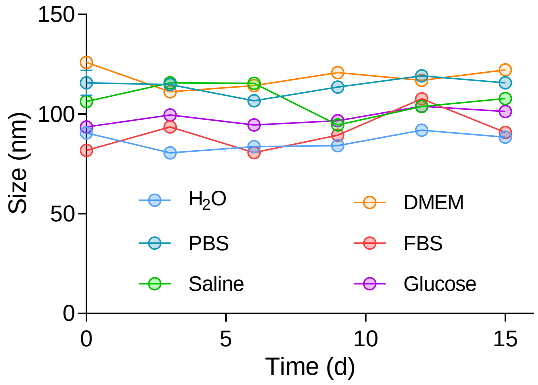

Supplementary Fig. 14 The hydrodynamic size profiles of PMNSG in different buffer solutions (H_2O, PBS, Saline, DMEM, FBS (60%) and Glucose). Source data are provided as a Source Data file.

Supplementary Table 4. Characterization of PMNSG in different physiological media. Source data are provided as a Source Data file.

Media	Size (nm)	Polydispersity index (PDI)
H_2O	90.6 ± 0.6	0.204 ± 0.010
PBS	115.7 ± 6.3	0.274 ± 0.002
Saline	106.3 ± 1.0	0.280 ± 0.013
Glucose	93.6 ± 0.4	0.183 ± 0.017
DMEM	125.9 ± 0.4	0.205 ± 0.011
FBS	81.8 ± 0.6	0.303 ± 0.024

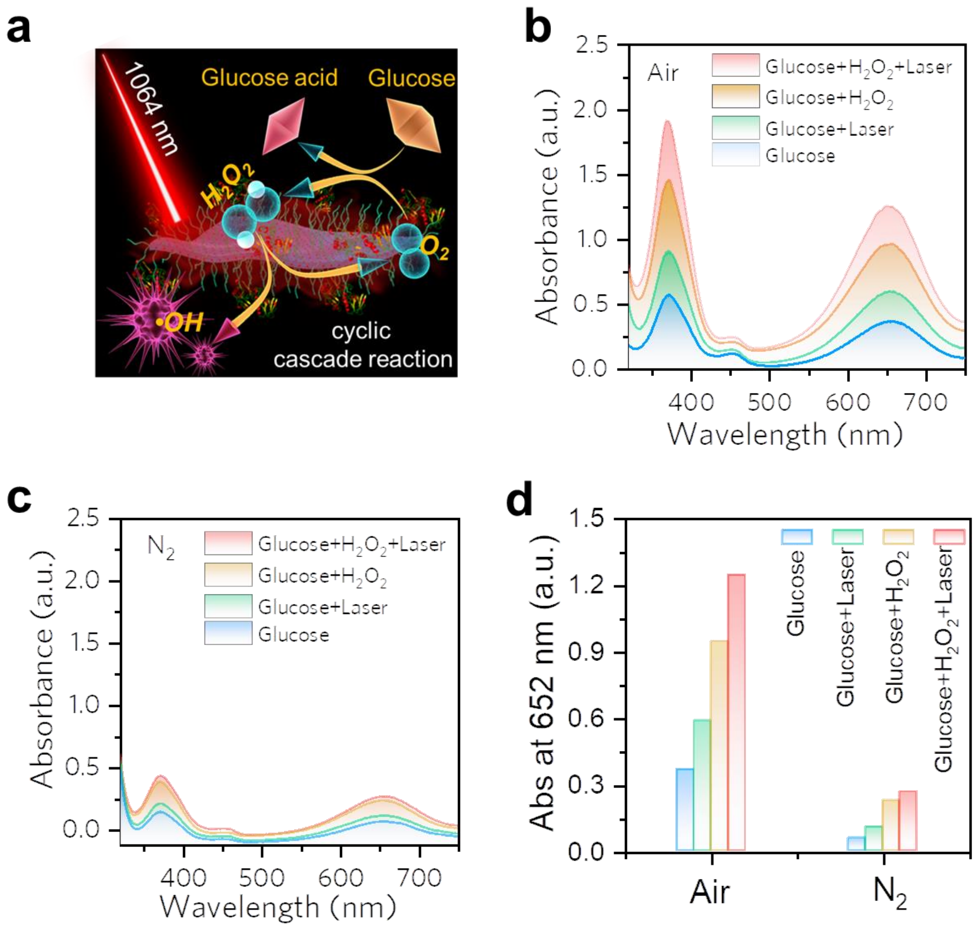

Supplementary Fig. 15 (a) Schematic illustration of the photothermal enhanced cyclic cascade reaction of PMNS under 1064-nm laser irradiation. Absorption spectra of TMB after different treatments in PMNSG solution under **(b)** normoxic and **(c)** hypoxic conditions (pre-saturated N_2). **(d)** Absorption intensity of TMB at 652 nm after different treatments in PMNSG solutions. Source data are provided as a Source Data file.

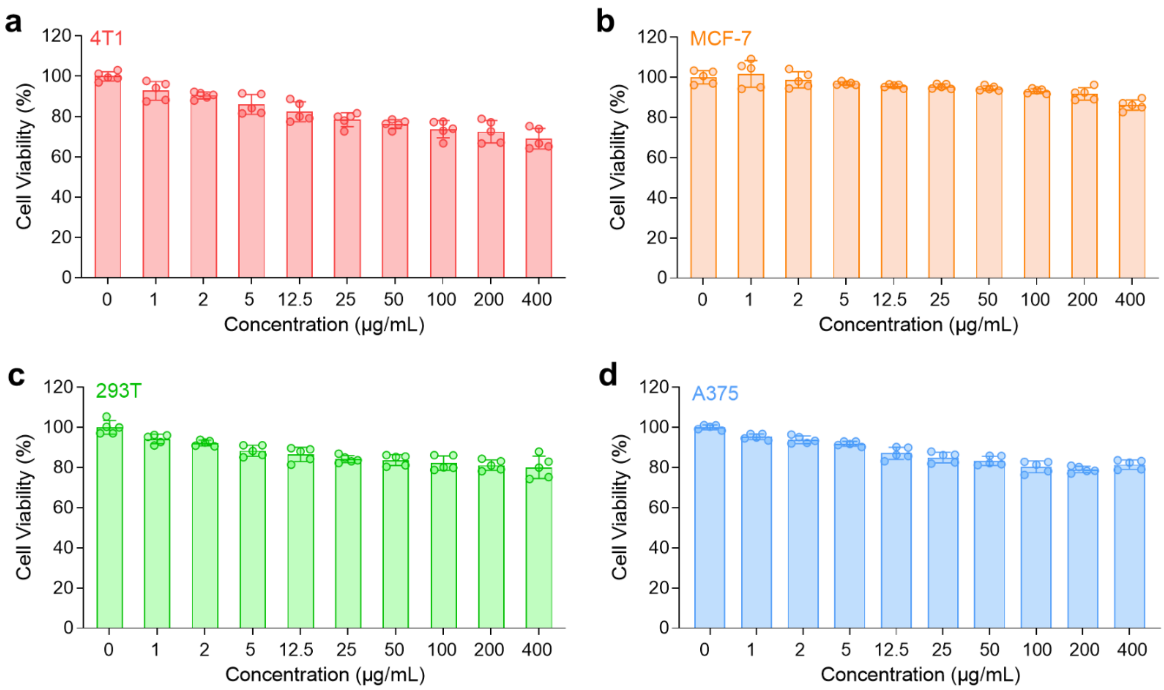

Supplementary Fig. 16 Cell viability of 4T1 cells **(a),** MCF-7 cells **(b),** 293-T cells **(c),** and A375 cells **(d)** after treated with PMNS for 24 h. Data are presented as mean ± SD. (n = 5). Source data are provided as a Source Data file.

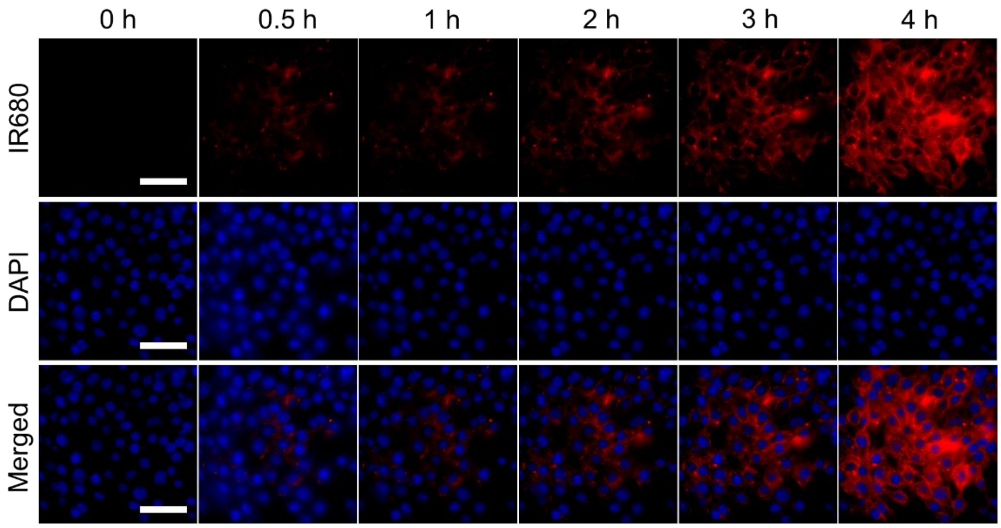

Supplementary Fig. 17 The real-time cells uptake of IR680 labeled PMNSG (0 - 4 h). Scale bar is 50 μm.

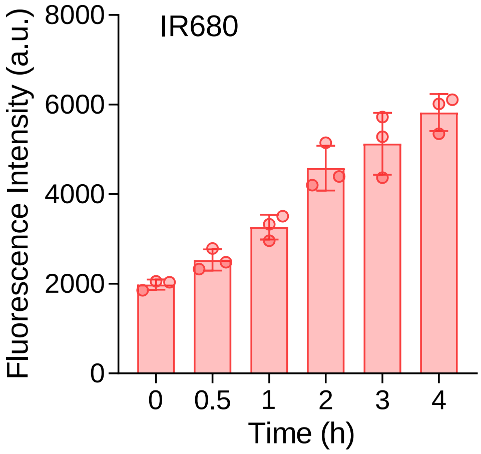

Supplementary Fig. 18 Quantification of red fluorescence signals (IR680) from **Supplementary Fig. 17**. Data are presented as mean ± SD. (n = 3). Source data are provided as a Source Data file.

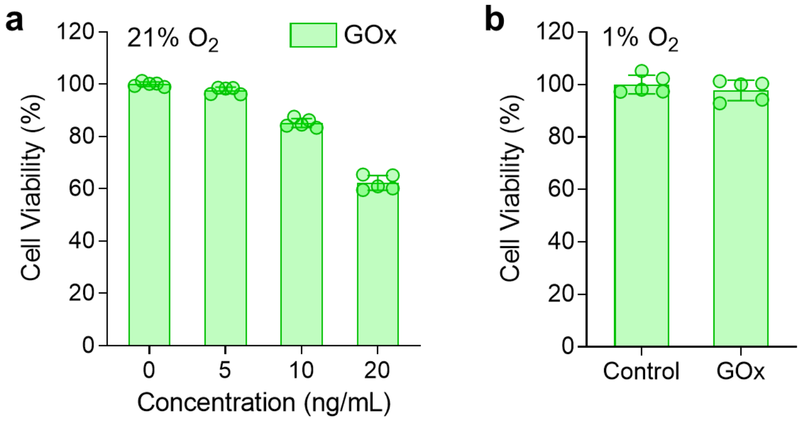

Supplementary Fig. 19 (a) Cell viability of 4T1 cells after treated with various concentrations of GOx (equivalent with that of conjugating on PMNS) under normoxic condition. **(b)** Cell viability of 4T1 cells after treated with PBS and GOx under hypoxic condition ($N_2/CO_2/O_2$: 94/5/1 in volume ratio). Data are presented as mean ± SD. (n = 5). Source data are provided as a Source Data file.

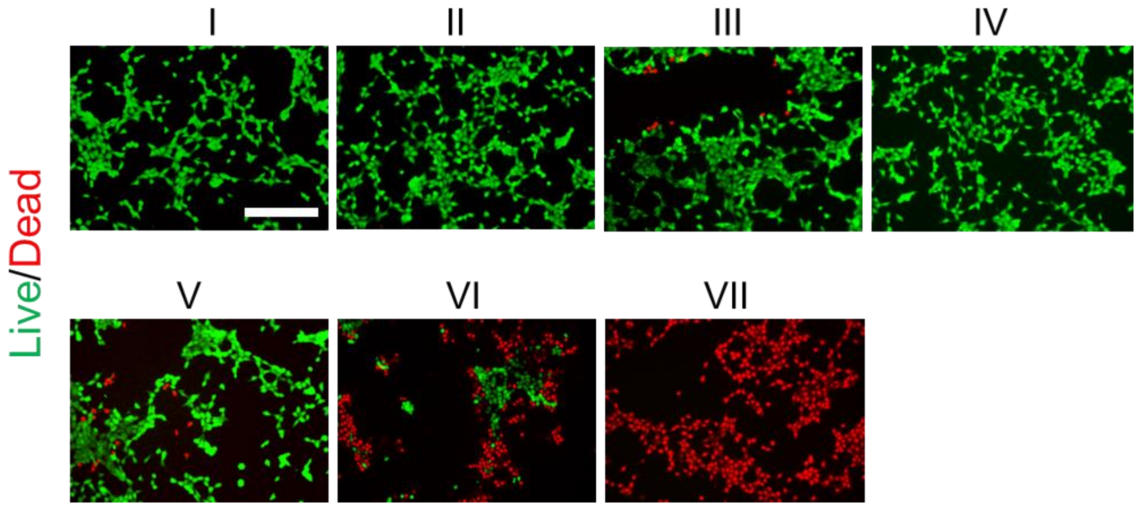

Supplementary Fig. 20 Fluorescence images of 4T1 cells co-staining with calcein-AM (live cell, green) and propidium iodide (dead cell, red) after different treatments (I: PBS, II: Laser, III: GOx, IV: PMNS, V: PMNS + Laser, VI: PMNSG and VII: PMNSG + Laser). n=3 independent experiments. Scale bar is 200 μm.

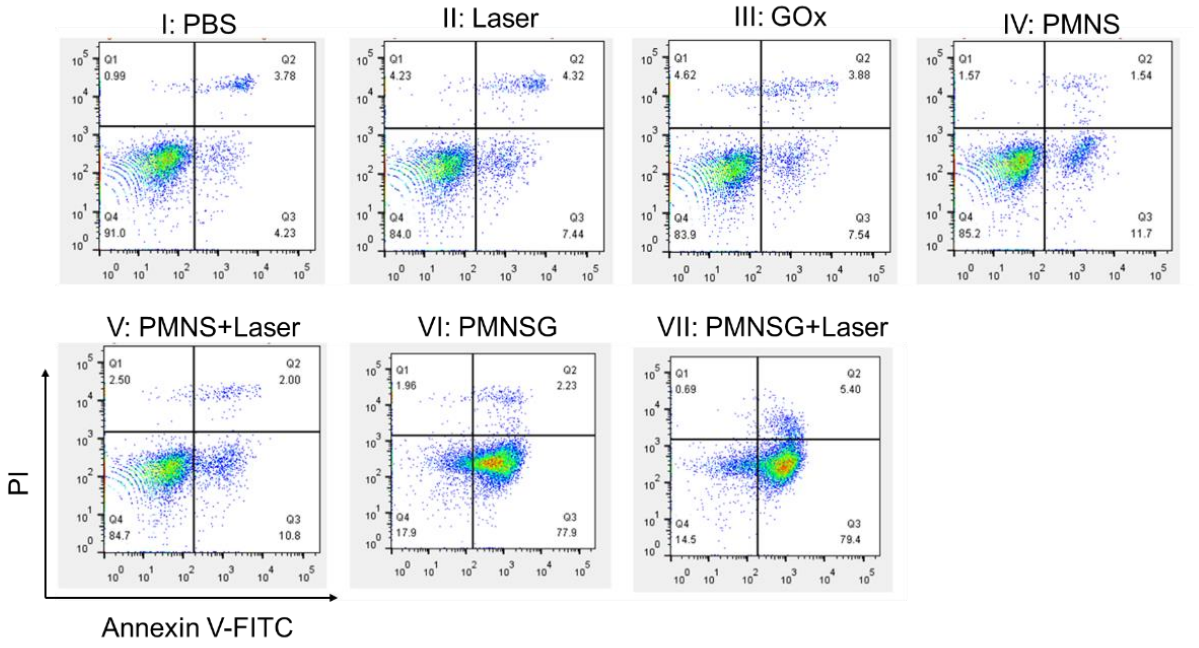

Supplementary Fig. 21 Annexin V-FITC/PI analyses (FACS gating strategy) of 4T1 cells after different treatments (PBS, Laser, GOx, PMNS, PMNS + Laser, PMNSG and PMNSG + Laser).

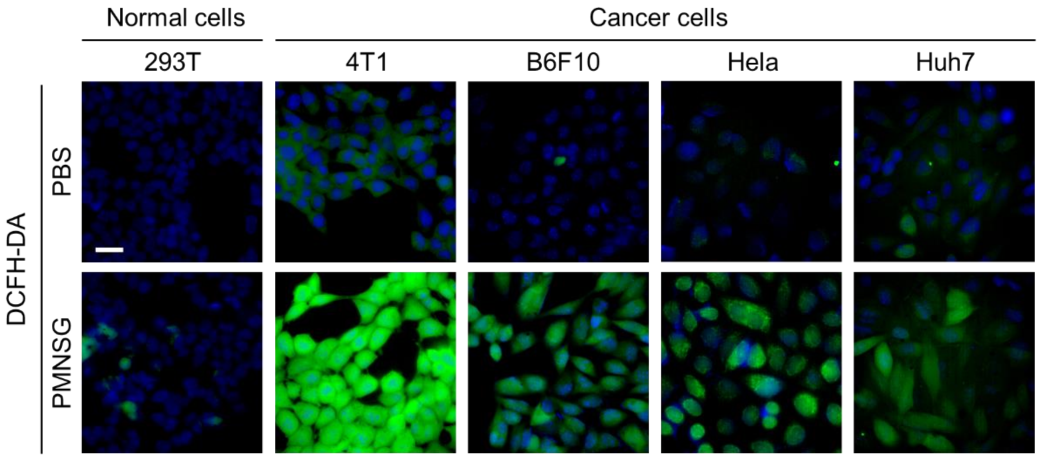

Supplementary Fig. 22 High content images of different types of cancer cells and normal cells after incubation with PBS or PMNSG for 12 h. ROS was indicated by green fluorescence from DCF-DA staining. Nuclei were stained with 4′,6-diamidine-2′-phenylindole dihydrochloride (DAPI) and indicated by blue fluorescence. 293T: human embryonic kidney cell line; 4T1: murine mammary carcinoma cell line; B6F10: murine melanoma cell line; Hela: human cervical carcinoma cell line; Huh7: human hepatoma cell line. n=3 independent experiments. Scale bar is 20 μm

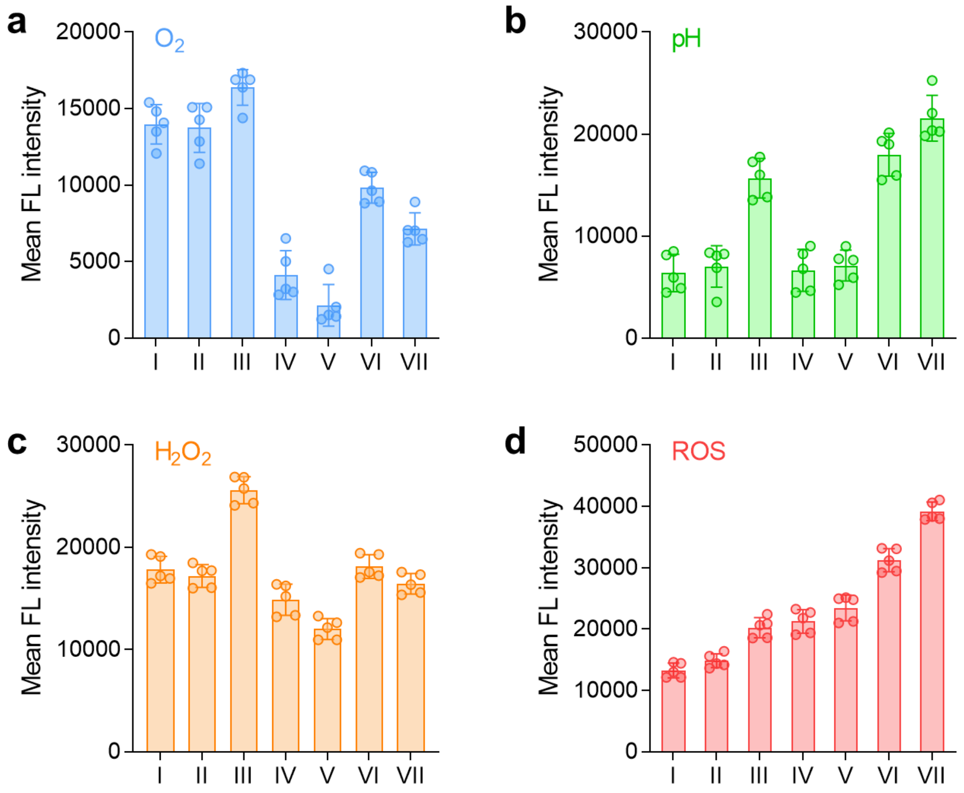

Supplementary Fig. 23 Quantification of fluorescence signals RDPP **(a),** BCECF-AM **(b),** Amplex Red **(c)** and DCFH-DA **(d)** after different treatments (I: PBS; II: Laser; III: GOx; IV: PMNS; V: PMNS + Laser; VI: PMNSG; VII: PMNSG + Laser). Data are presented as mean ± SD. (n = 5). Source data are provided as a Source Data file.

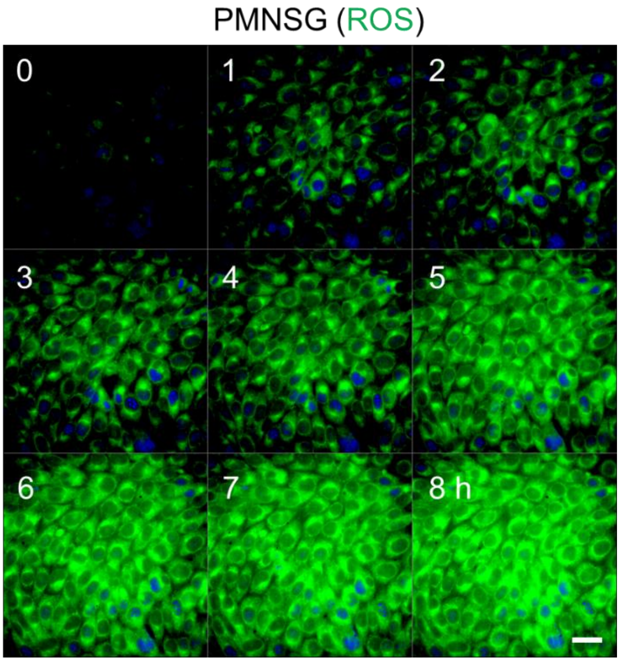

Supplementary Fig. 24 Real-time high content images of 4T1 cells stained with DCFH-DA after PMNSG treatment. n=3 independent experiments. Scale bar is 20 μm.

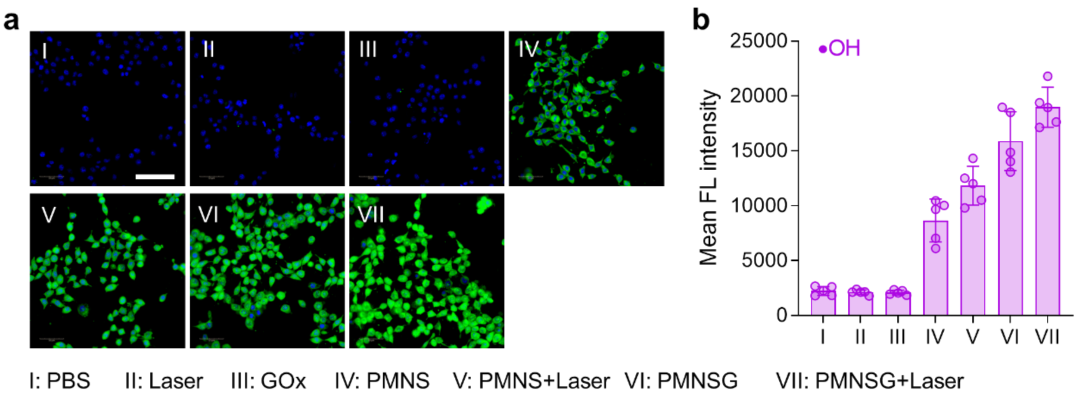

Supplementary Fig. 25 (a) High content images of 4T1 cells stained with HPF after different treatments (scale bar is 50 μm) and **(b)** the corresponding quantification of fluorescence signals. Data are presented as mean ± SD (n = 5). Source data are provided as a Source Data file.

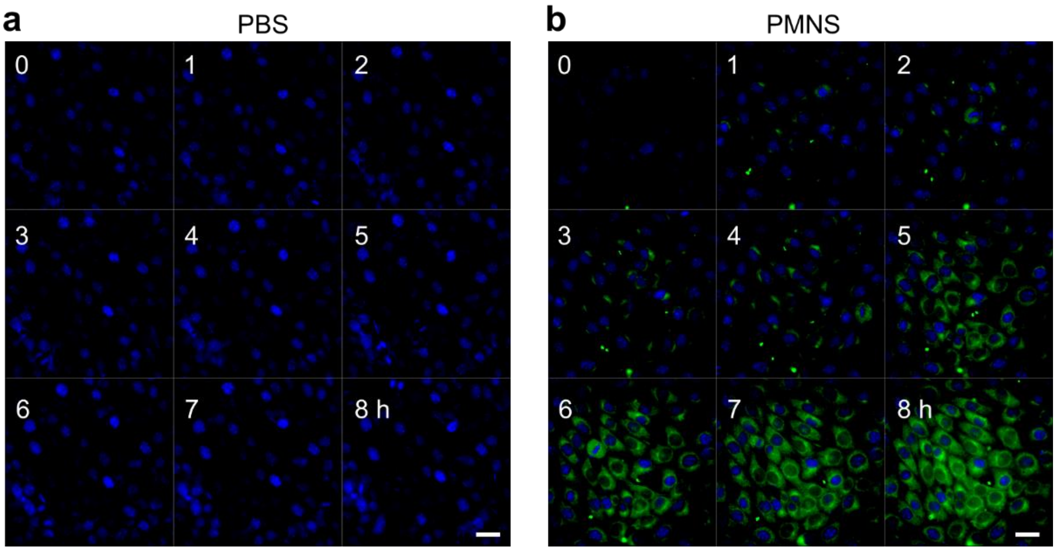

Supplementary Fig. 26 Real-time high content images of 4T1 cells stained with HPF after **(a)** PBS and **(b)** PMNS treatments. n=3 independent experiments. Scale bar is 20 μm.

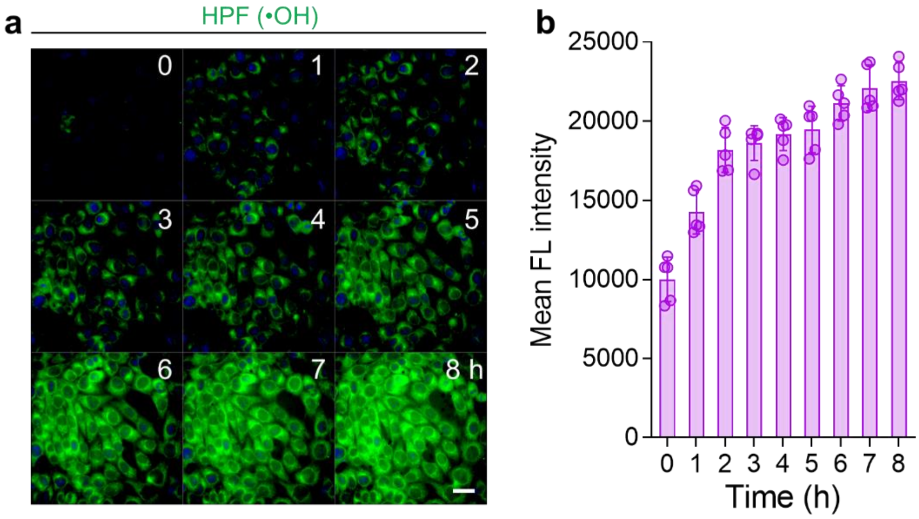

Supplementary Fig. 27 (a) The evolution of •OH in 4T1 cells incubated with PMNSG. **(b)** Quantification of green fluorescence signals from the green fluorescent •OH probe hydroxyphenyl fluorescein (HPF). Data are presented as mean ± SD. (n = 5). Source data are provided as a Source Data file.

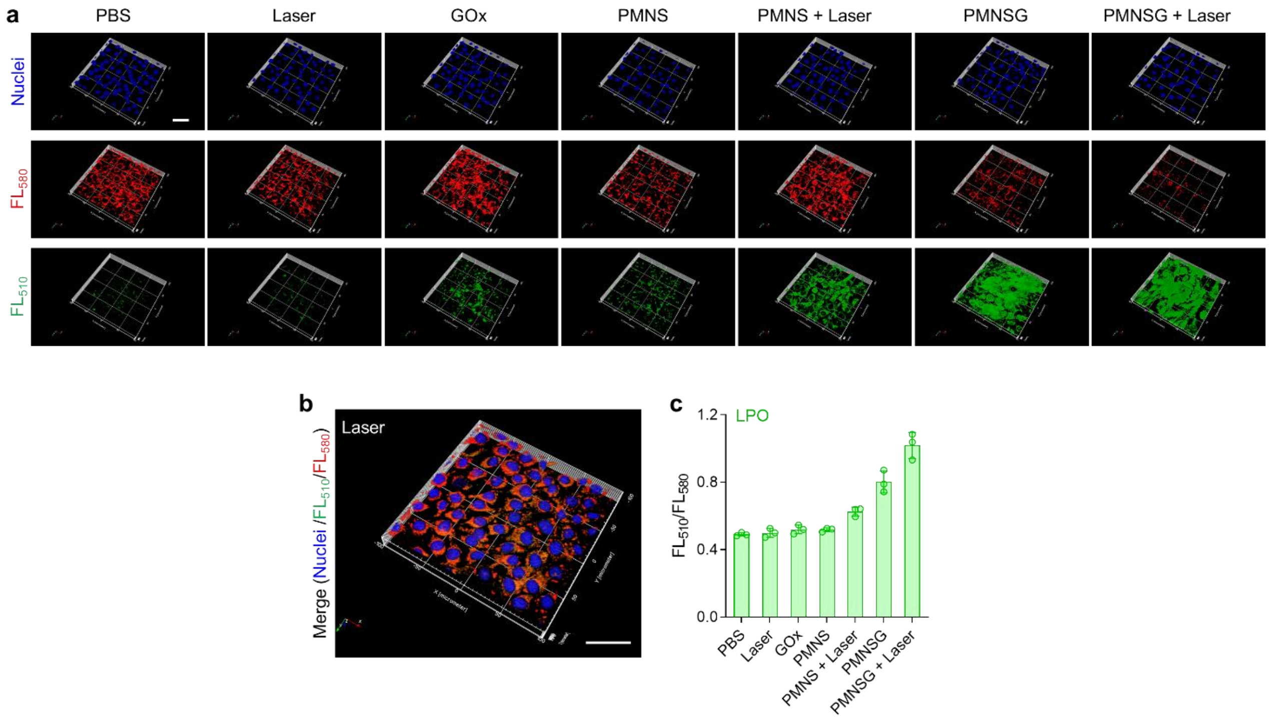

Supplementary Fig. 28 (**a**) Evaluation of LPO in 4T1 cells after various treatments (PBS; Laser; GOx; PMNS; PMNS + laser; PMNSG; PMNSG + laser). Scale bar is 50 μm. (**b**) Merged high content image of 4T1 cells after laser irradiation alone. Scale bar is 50 μm. (**c**) Quantification of fluorescence intensity ratio of FL_{510}/FL_{580} from (**a**) under different treating conditions. Data are presented as mean ± SD. (n = 3). Source data are provided as a Source Data file.

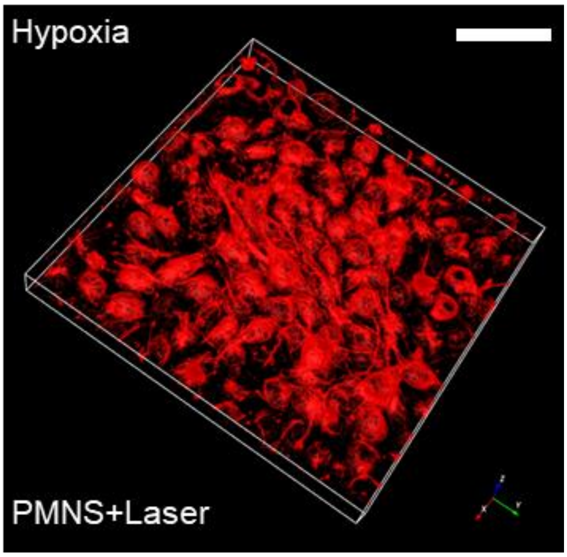

Supplementary Fig. 29 Immunofluorescence imaging of 4T1 cells with stained HIF-1α (green) and tubulin (red) after treated PMNS + Laser under hypoxic condition (1% O_2, 5%CO_2, and 94% N_2). Scale bar is 50 μm

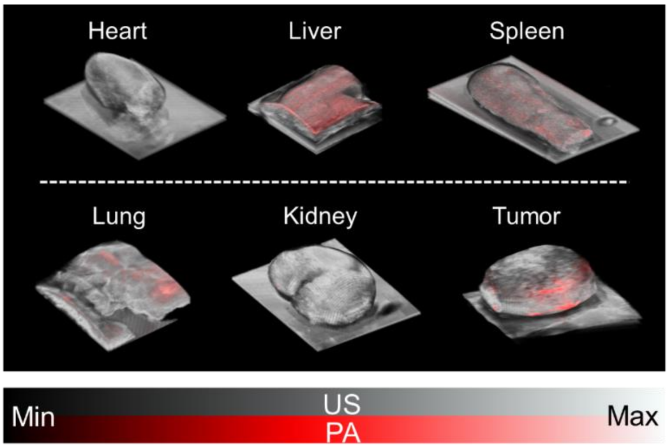

Supplementary Fig. 30 Ex vivo 3D-rendered PA/US images of various organs and tumor at 24 h post injection of PMNS.

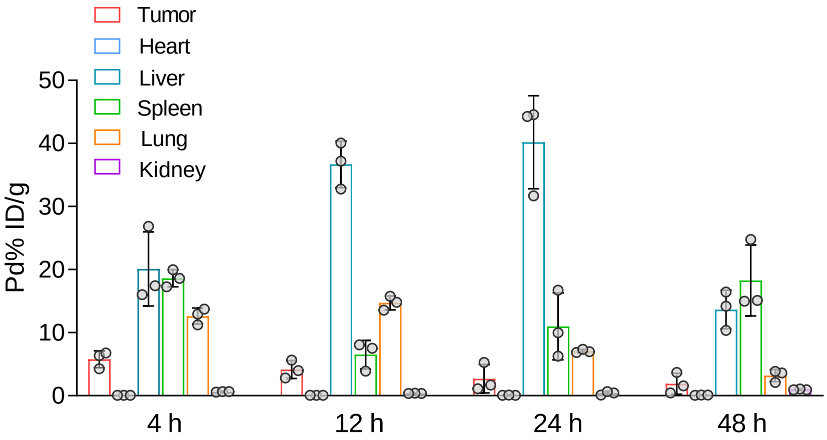

Supplementary Fig. 31 Ex vivo biodistribution of PMNSG in tumor and major organs of mice bearing 4T1 tumor at 4, 12, 24, and 48 h. Data are presented as mean ± SD (n = 3). Source data are provided as a Source Data file.

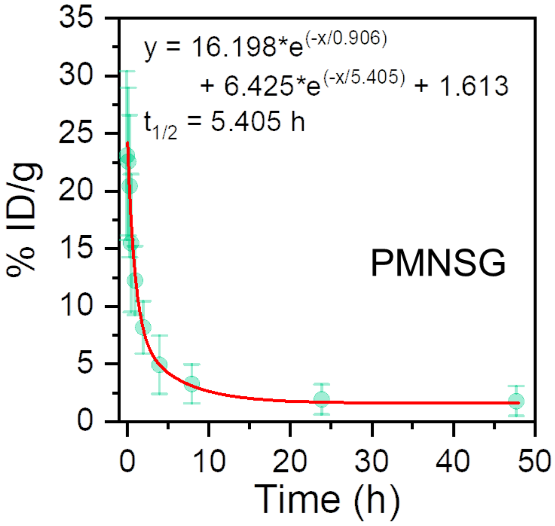

Supplementary Fig. 32 Blood circulation of PMNSG by measuring Pd concentrations over a span of 48 h after intravenous injection. Data are presented as mean ± SD (n = 3). Source data are provided as a Source Data file.

The hemolysis was very low even at a high concentrations (500 μg mL^{-1}) of PMNSG incubation (Supplementary Fig.33). Furthermore, the histological examination of various major organs (heart, liver, spleen, lung and kidney) showed no pathological abnormality or inflammation after treated with PMNSG ((Supplementary Fig. 34). Besides these, the PMNSG exhibited a biodegradation behavior under neutral (pH = 7.4) simulated body fluid (SBF) mediums (Supplementary Fig. 35)[8]. These results demonstrated that the PMNSG possessed good biosafety and biodegradability.

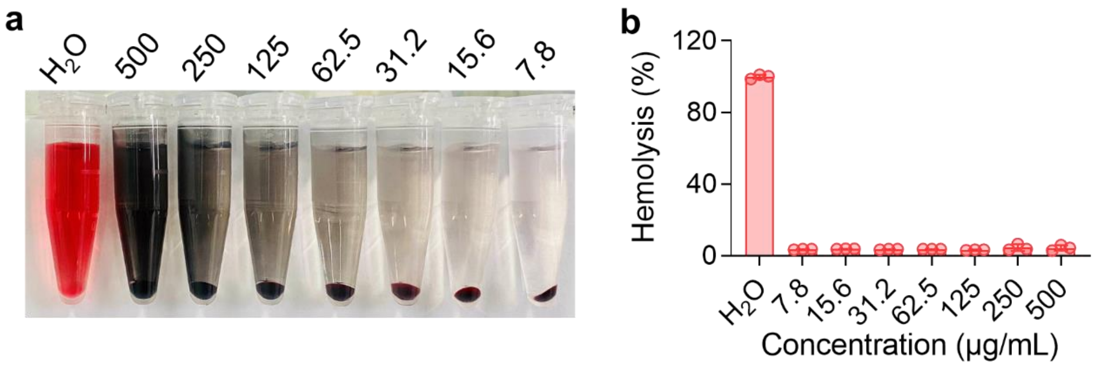

Supplementary Fig. 33 Hemolysis of PMNSG solution at various concentrations (the mixtures were centrifuged to detect the presence of hemoglobin in the supernatants visually). Data are presented as mean ± s.e.m. (n = 3). Source data are provided as a Source Data file.

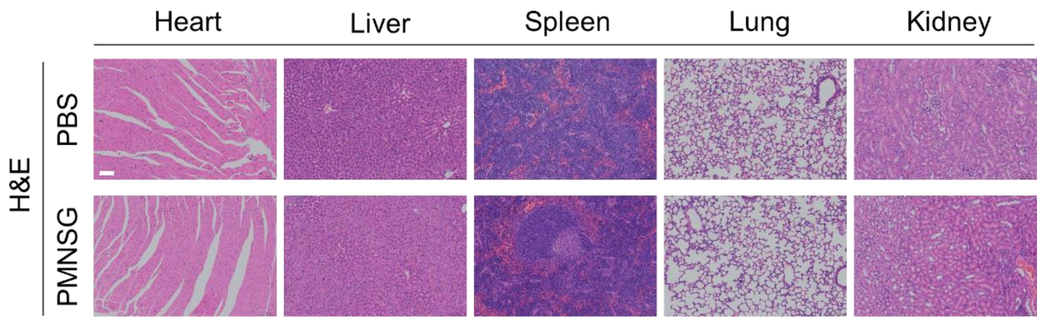

Supplementary Fig. 34 Histological examination (H&E staining) of slices for major organs various major organs (heart, liver, spleen, lung and kidney) after different treatments. n=3 independent experiments. Scale bar is 100 μm.

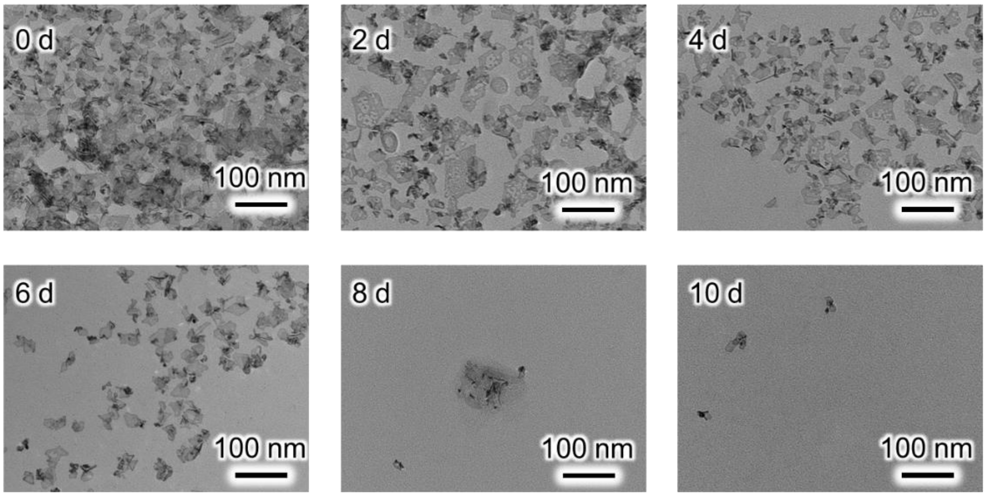

Supplementary Fig. 35 Degradability of PMNSG. TEM images of PMNSG after incubation in simulated body fluid (SBF) mediums (pH = 7.4) at 37 ^{o}C for different durations.

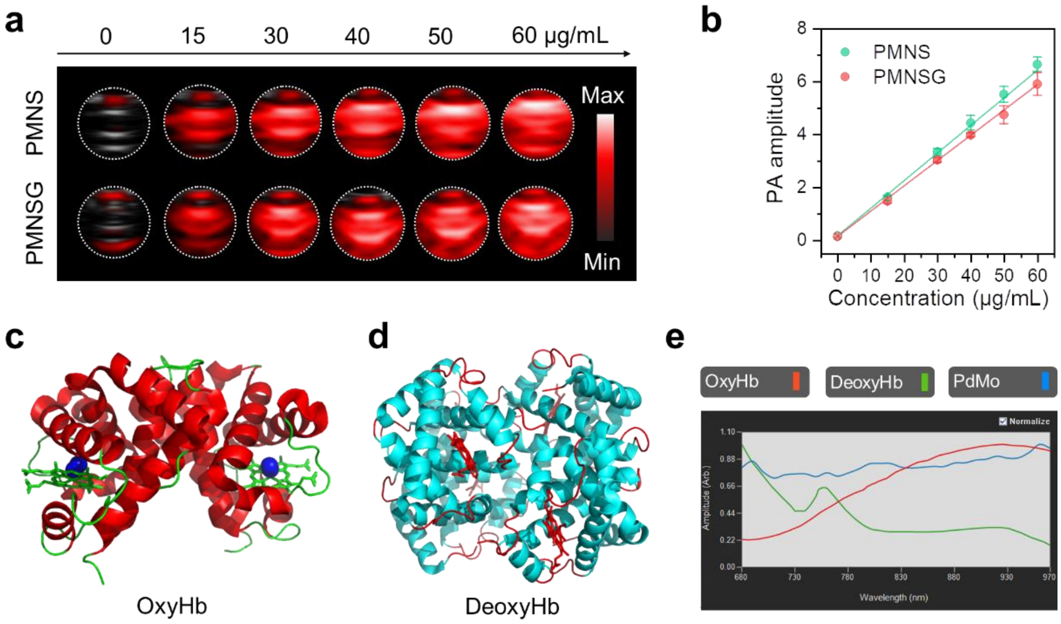

Supplementary Fig. 36 (a) In vitro NIR-I PA images of PMNS and PMNSG at different concentrations and **(b)** the corresponding liner fits of NIR-I PA amplitude as a function of concentration at 780 nm, respectively. Data are presented as mean ± SD. (n = 3). Structure of OxyHb (PDB: 1HHO) **(c)** and DeoxyHb (PDB: 2HHB) **(d). (e)** Normalized PA spectra of OxyHb, DeoxyHb (the spectra are obtained from Vevo LAZR-X PA imaging system) and PdMo, respectively. Source data are provided as a Source Data file.

The sO_2 value of control group showed almost no changes at about 21.7%, consistent with a solid 4T1 tumor with hypoxic property. After the treatment of PMNS, the sO_2 value rapidly increased and reached its maximum of 46.8% at the time point of 4 h within the tumor regions. For the PMNSG treatment group, the sO_2 value also exhibited an increasing trend and possessed a maximum sO_2 value of 39.7%, which is lower than that of the PMNS treatment group. These results demonstrated the efficient oxygen supply ability of PMNS for the aerobic catalysis of GOx toward glucose.

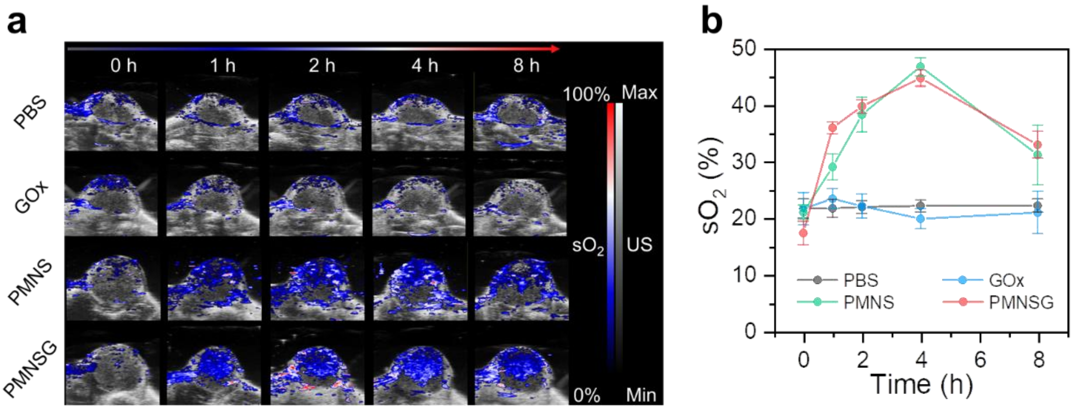

Supplementary Fig. 37 Time-course of PA images of sO_2 in tumor region after i.v. injection of PBS, GOx, PMNS and PMNSG **(a)** and the corresponding quantification of sO_2 value as a function of time, respectively **(b).** Data are presented as mean ± SD. (n = 5). Source data are provided as a Source Data file.

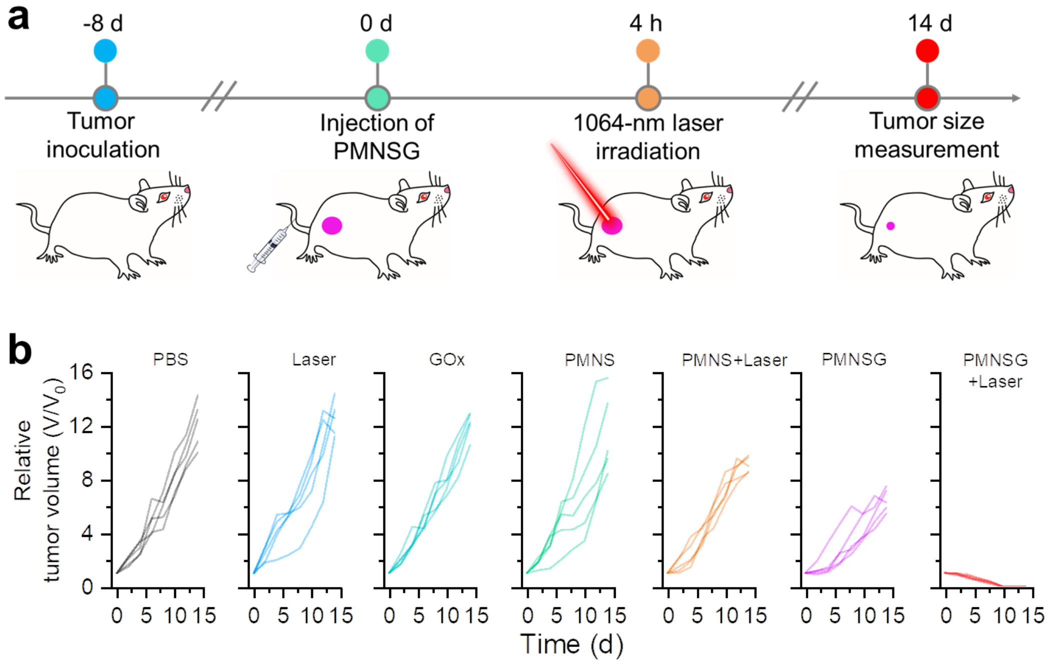

Supplementary Fig. 38 (a) Schematic of therapeutic approach for tumor-bearing mice. (b) Individual tumor growth curves of all groups after various treatments (PBS, Laser, GOx, PMNS, PMNS + Laser, PMNSG and PMNSG + Laser). Source data are provided as a Source Data file.

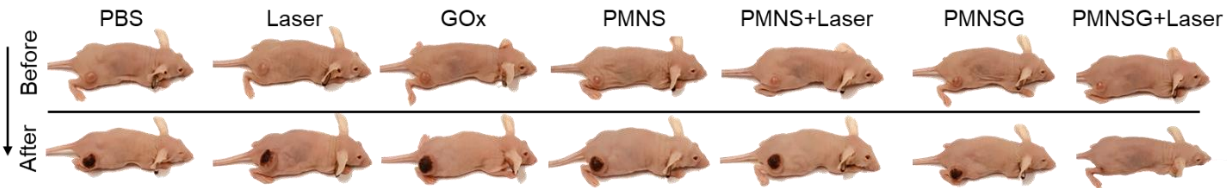

Supplementary Fig. 39 Representative photos of the 4T1 tumor-bearing mice with different treatments.

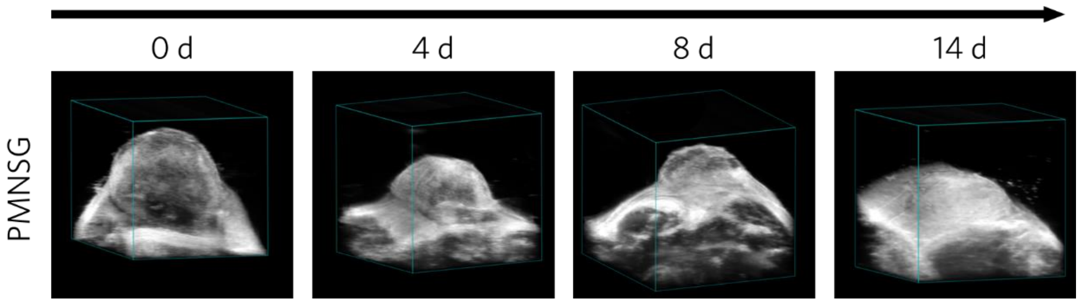

Supplementary Fig. 40 B-mode US images of tumor region for PMNSG treatment group.

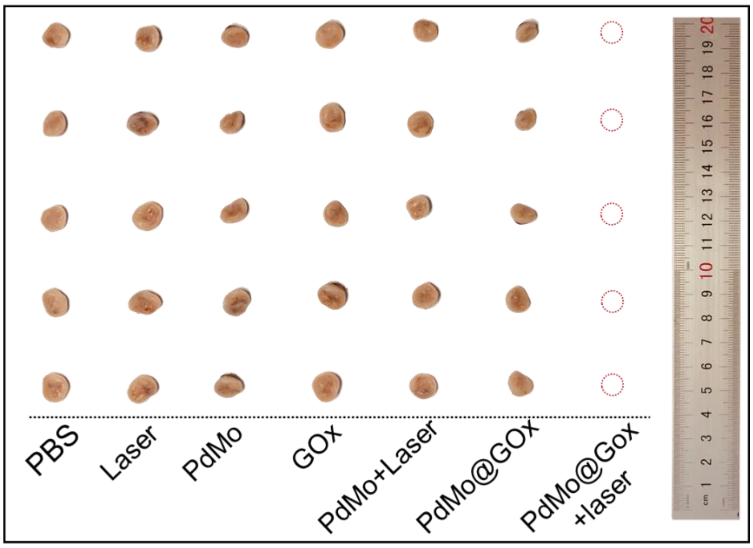

Supplementary Fig. 41 Photographic images of tumors excised from different groups after various treatments indicated.

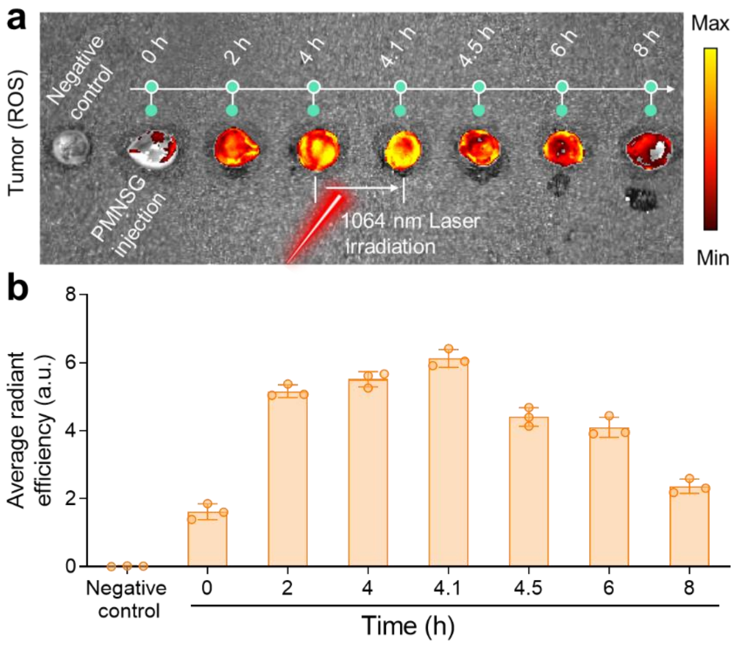

Supplementary Fig. 42 Bioluminescence images **(a)** and the corresponding quantitative analysis of tumor tissues that were stained with DCFH-DA **(b).** Data are presented as mean ± SD. (n = 3). Source data are provided as a Source Data file.

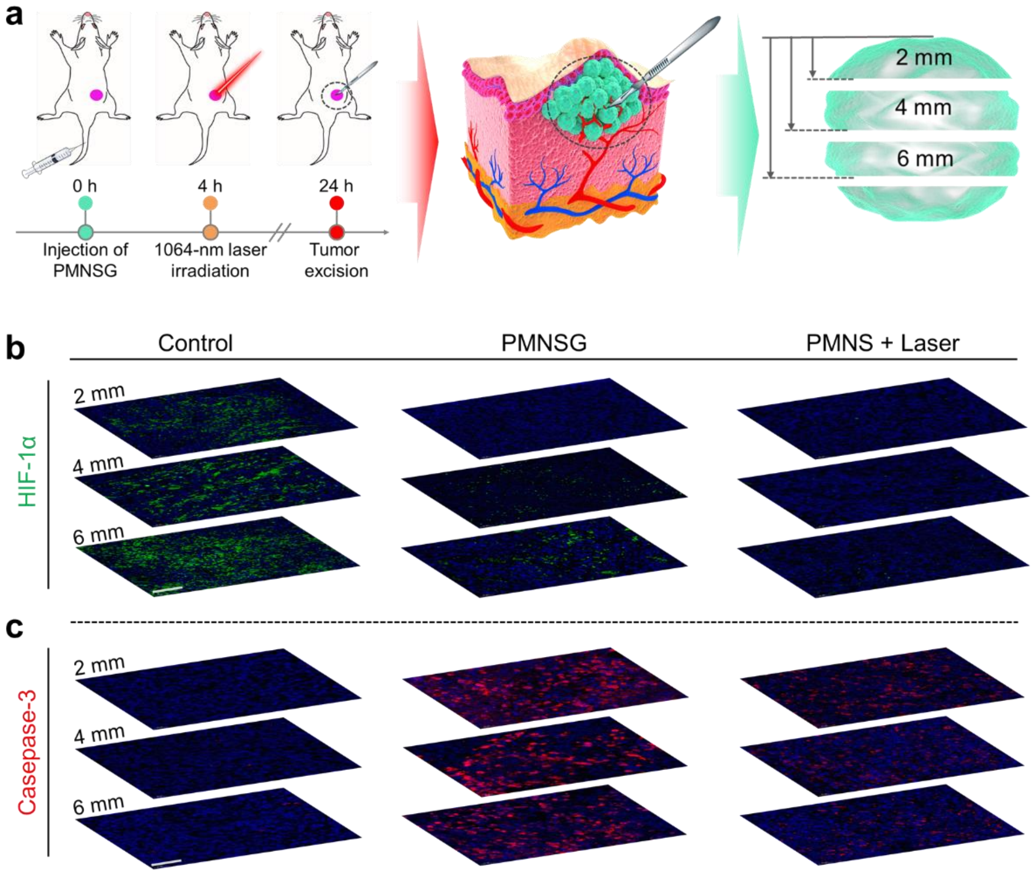

Supplementary Fig. 43 (a) Scheme of mild 1064-nm laser irradiated depths of tumor. Immunofluorescence staining images of **(b)** HIF-1α and **(c)** caspase-3 for tumor slices at different tumor depth after PBS treatments. Scale bar is 50 μm.

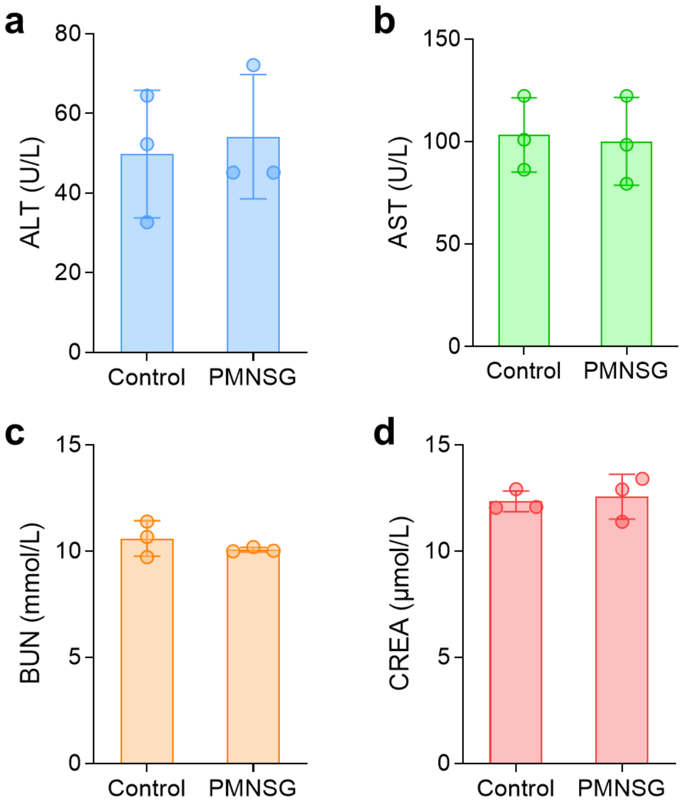

Supplementary Fig. 44 Blood biochemistry analysis (ALT, AST, BUN, and CREA) of healthy mice after intravenously injected with saline or PMNSG for 14 days. Data are presented as mean ± SD. (n = 3). Source data are provided as a Source Data file.

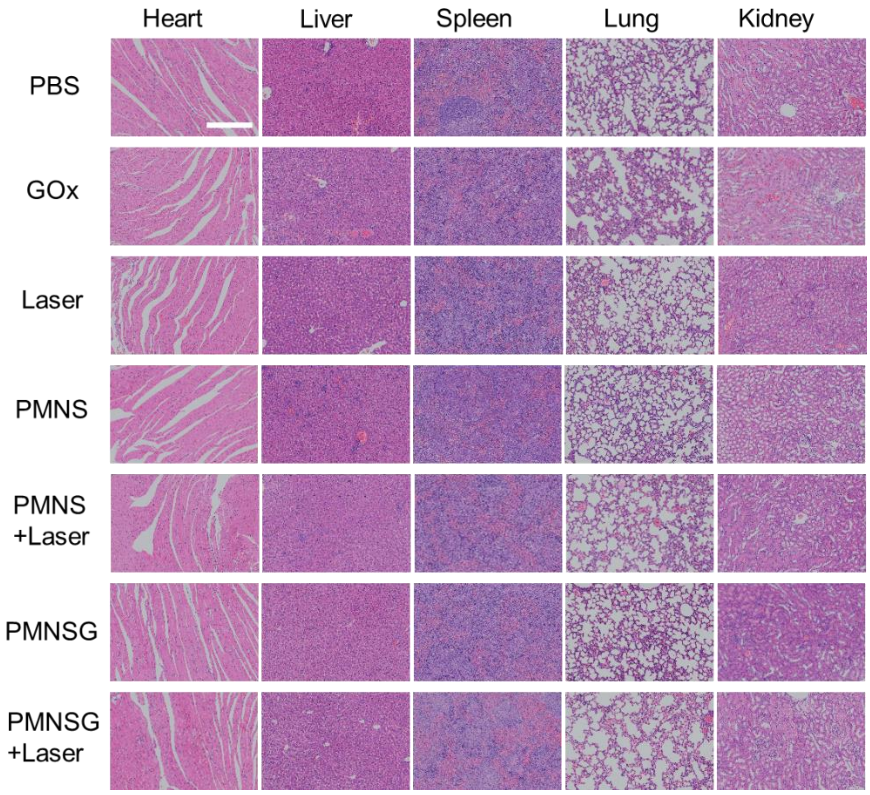

Supplementary Fig. 45 H&E staining of major organs collected from different groups after various treatments (PBS, Laser, GOx, PMNS, PMNS + Laser, PMNSG and PMNSG + Laser) indicated. n=3 independent experiments. Scale bar is 100 μm.

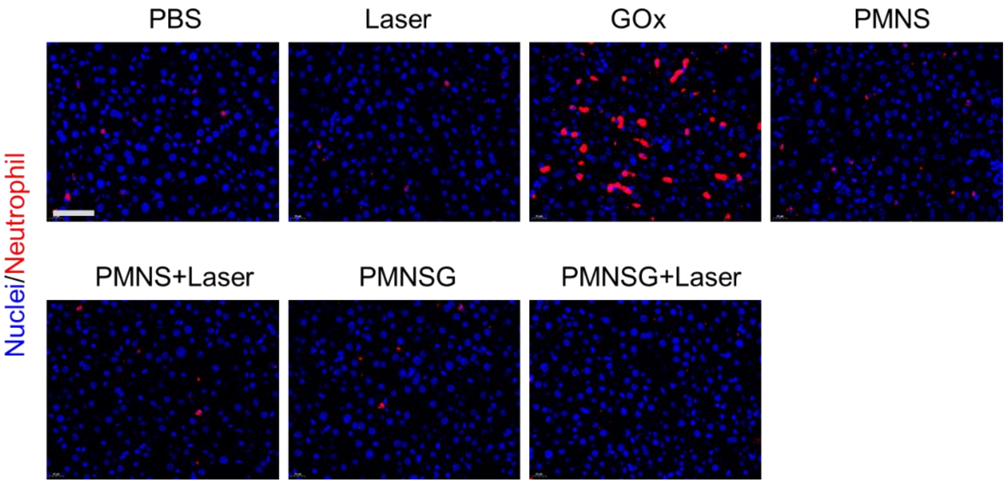

Supplementary Fig. 46 Immunofluorescence staining of infiltration neutrophils (Ly6G^{+}) of liver slices. n=3 independent experiments. Scale bar is 50 μm.

References

1. Zhang H*, et al.* Photoreductive synthesis of water-soluble fluorescent metal nanoclusters. *Chem. Commun.* **48**, 567-569 (2012).
2. Takeda M, Suzuki J. Crystallographic heterodyne phase detection for highly sensitive lattice-distortion measurements. *J. Opt. Soc. Am. A* **13**, 1495-1500 (1996).
3. Hÿtch MJ, Snoeck E, Kilaas R. Quantitative measurement of displacement and strain fields from HREM micrographs. *Ultramicroscopy* **74**, 131-146 (1998).
4. Lei S, Chen J, Zeng K, Wang M, Ge X. Visual dual chemodynamic/photothermal therapeutic nanoplatform based on superoxide dismutase plus Prussian blue. *Nano Res.* **12**, 1071-1082 (2019).
5. Xi Z*, et al.* Strain effect in palladium nanostructures as nanozymes. *Nano Lett.* **20**, 272-277 (2020).
6. Long R*, et al.* Surface facet of palladium nanocrystals: a key parameter to the activation of molecular oxygen for organic catalysis and cancer treatment. *J. Am. Chem. Soc.* **135**, 3200-3207 (2013).
7. Luo M*, et al.* PdMo bimetallene for oxygen reduction catalysis. *Nature* **574**, 81-85 (2019).
8. Huo M, Wang L, Chen Y, Shi J. Tumor-selective catalytic nanomedicine by nanocatalyst delivery. *Nat. Commun.* **8**, 357 (2017).
