# Supplementary material for: In vivo three-dimensional multispectral photoacoustic imaging of dual enzyme-driven cyclic cascade reaction for tumor catalytic therapy
Source: Nat Commun. 2022 Mar 11;13:1298. doi: 10.1038/s41467-022-29082-1 (PMC8917194; doi:10.1038/s41467-022-29082-1)
Supplement: Supplementary file 3 — Reporting Summary [file 41467_2022_29082_MOESM3_ESM.pdf]

nature portfolio

Corresponding author(s): Peng Huang

Last updated by author(s): Jan 27, 2022

Reporting Summary

Nature Portfolio wishes to improve the reproducibility of the work that we publish. This form provides structure for consistency and transparency in reporting. For further information on Nature Portfolio policies, see our Editorial Policies and the Editorial Policy Checklist.

Statistics

For all statistical analyses, confirm that the following items are present in the figure legend, table legend, main text, or Methods section.

n/a	Confirmed	
☐	☒	The exact sample size (*n*) for each experimental group/condition, given as a discrete number and unit of measurement
☐	☒	A statement on whether measurements were taken from distinct samples or whether the same sample was measured repeatedly
☐	☒	The statistical test(s) used AND whether they are one- or two-sided<br>*Only common tests should be described solely by name; describe more complex techniques in the Methods section.*
☐	☒	A description of all covariates tested
☐	☒	A description of any assumptions or corrections, such as tests of normality and adjustment for multiple comparisons
☐	☒	A full description of the statistical parameters including central tendency (e.g. means) or other basic estimates (e.g. regression coefficient) AND variation (e.g. standard deviation) or associated estimates of uncertainty (e.g. confidence intervals)
☐	☒	For null hypothesis testing, the test statistic (e.g. *F*, *t*, *r*) with confidence intervals, effect sizes, degrees of freedom and *P* value noted<br>*Give P values as exact values whenever suitable.*
☒	☐	For Bayesian analysis, information on the choice of priors and Markov chain Monte Carlo settings
☒	☐	For hierarchical and complex designs, identification of the appropriate level for tests and full reporting of outcomes
☒	☐	Estimates of effect sizes (e.g. Cohen's *d*, Pearson's *r*), indicating how they were calculated

Our web collection on statistics for biologists contains articles on many of the points above.

Software and code

Policy information about availability of computer code

Data collection	UV-Vis-NIR data collection: UV Winlab (Version 7.1)<br>High Resolution Transmission Electron Microscope: Gatan Microscopy Suite (Version 3.0)<br>Flow Cytometry: CellQuest Pro (version 5.1)<br>Photoacoustic ultrasound image recording: VisualSonics Vevo LAZR-X data collection software (Version 3.2.0)<br>High content images: Operetta CLS_Harmony(version 4.9)<br>In vivo fluorescence images: PerkinElmer Living Imaging Software (version 4.5)
Data analysis	Microsoft Excel (version 2019); Graphpad Prism (version 8.0); FlowJo_V10 (version 10.7.1); Vevo LAB (version 3.2.0); OriginPro 2018b; Gatan Microscopy Suite (Version 3.0); Living image software (version 4.5)

For manuscripts utilizing custom algorithms or software that are central to the research but not yet described in published literature, software must be made available to editors and reviewers. We strongly encourage code deposition in a community repository (e.g. GitHub). See the Nature Portfolio guidelines for submitting code & software for further information.

Data

Policy information about availability of data

All manuscripts must include a data availability statement. This statement should provide the following information, where applicable:

- Accession codes, unique identifiers, or web links for publicly available datasets
- A description of any restrictions on data availability
- For clinical datasets or third party data, please ensure that the statement adheres to our policy

The authors declare that the data supporting the findings of this study are available within the article and its Supplementary Information. Additional data are available from the corresponding author upon request. Source data are provided with this paper.

Field-specific reporting

Please select the one below that is the best fit for your research. If you are not sure, read the appropriate sections before making your selection.

☒ Life sciences ☐ Behavioural & social sciences ☐ Ecological, evolutionary & environmental sciences

For a reference copy of the document with all sections, see nature.com/documents/nr-reporting-summary-flat.pdf

Life sciences study design

All studies must disclose on these points even when the disclosure is negative.

Sample size	Sample sizes were based on a combination of the resource-equation method and prior laboratory experience, to ensure statistical and biological significance. One-way ANOVA with Tukey's multiple comparisons was used for multiple comparisons when more than two groups were compared, and one-tailed or two-tailed Student's t-test was used for two-group comparisons.
Data exclusions	No data were excluded from the analyses.
Replication	All experiments were successfully replicated at least in 2 independent experiments.
Randomization	Cells were randomly assigned to different groups and performed independently. Animals were randomly allocated to each group and performed independently
Blinding	The investigators were blinded to the cell groups and mouse groups when collecting the results.

Reporting for specific materials, systems and methods

We require information from authors about some types of materials, experimental systems and methods used in many studies. Here, indicate whether each material, system or method listed is relevant to your study. If you are not sure if a list item applies to your research, read the appropriate section before selecting a response.

Materials & experimental systems

n/a	Involved in the study	
☐	☒	Antibodies
☐	☒	Eukaryotic cell lines
☒	☐	Palaeontology and archaeology
☐	☒	Animals and other organisms
☒	☐	Human research participants
☒	☐	Clinical data
☒	☐	Dual use research of concern

Methods

n/a	Involved in the study	
☒	☐	ChIP-seq
☐	☒	Flow cytometry
☒	☐	MRI-based neuroimaging

Antibodies

Antibodies used

1. Monoclonal Anti-mouse α-Tubulin antibody, SigmaAldrich, cat no. T9026
2. Anti-rabbit IgG H&L (Alexa Fluor® 488), Abcam, cat no. ab150077
3. HIF-α (D1S7W) XP Rabbit mAb #36169, Cell Signaling Technology, cat no. 36169T
4. Anti-rabbit Caspase-3 antibody, Abcam, cat no.
5. Anti-Ki67 Rabbit pAb, G SERVICEBIO, cat no. GB111499
6. Anti-Ly6g Rabbit pAb, G SERVICEBIO, cat no. GB11229
7. Cleaved Caspase-3 (Asp175) Antibody #9661, Cell Signaling Technology, cat no. 9661S
8. GLUT1 (D3J3A) mAb #12939, Cell Signaling Technology, cat no. 12939S
9. Anti-VEGF antibody [EPR20705] Rabbit, Abcam, cat no. ab214424

	10. HIF-1α (D2U3T) Rabbit mAb #14179, Cell Signaling Technology, cat no. 14179S<br>11. β-Actin (D6A8) Rabbit mAb #8457, Cell Signaling Technology, cat no. 8457S<br>12. Caspase-3 Antibody #9662, Cell Signaling Technology, cat no.9662S
Validation	We have only used antibodies from commercial sources. The suppliers have a standard validation system to ensure reproducibility, and validation data are available online at the manufacturers' websites.<br>1. Monoclonal Anti-α-Tubulin antibody:<br>https://www.sigmaaldrich.cn/CN/zh/product/sigma/t9026?context=product#<br>2. Anti-rabbit IgG (H+L),F(ab)2 Fragment (Alexa Fluor 488 Conjugate):<br>https://www.abcam.com/goat-rabbit-igg-hl-alexa-fluor-488-ab150077.html<br>3. HIF-α (D1S7W) XP Rabbit mAb #36169:<br>https://www.cellsignal.com/products/primary-antibodies/hif-1a-d1s7w-xp-rabbit-mab/36169<br>4. Anti-Caspase-3:<br>https://www.abcam.com/caspase-3-antibody-ab44976.html<br>5. Anti-Ki67 Rabbit pAb:<br>https://www.servicebio.cn/goodsdetail?id=3931<br>6. Anti-Ly6g Rabbit pAb:<br>https://www.servicebio.cn/goodsdetail?id=1454<br>7. Cleaved Caspase-3 (Asp175) Antibody #9661:<br>https://www.cellsignal.cn/products/primary-antibodies/cleaved-caspase-3-asp175-5a1e-rabbit-mab/966<br>8. GLUT1 (D3J3A) Rabbit mAb #12939:<br>https://www.cellsignal.cn/products/primary-antibodies/glut1-d3j3a-rabbit-mab/12939<br>9. Anti-VEGF antibody:<br>https://www.abcam.cn/vegfa-antibody-epr20705-ab214424.html<br>10. HIF-1α (D2U3T) Rabbit mAb #14179:<br>https://www.cellsignal.cn/products/primary-antibodies/hif-1a-d2u3t-rabbit-mab/14179<br>11. β-Actin (D6A8) Rabbit mAb #8457:<br>https://www.cellsignal.cn/products/primary-antibodies/b-actin-d6a8-rabbit-mab/8457<br>12. 6. Caspase-3 Antibody #9662:<br>https://www.cellsignal.cn/products/primary-antibodies/caspase-3-antibody/9662

Eukaryotic cell lines

Policy information about cell lines

Cell line source(s)	4T1 (mouse mammary carcinoma), MCF-7 (human mammary carcinoma), A375 (human malignant melanoma) and 293T (human embryonic kidney), B6F10 (murine melanoma), Huh7 (human hepatoma) and Hela (human cervical carcinoma) cell lines were obtained from the Cell Bank of the Chinese Academy of Sciences (Shanghai, China).
Authentication	All cell lines used in this study were authenticated by the Cell Bank of the Chinese Academy of Science. After receiving cell lines, cell identity was confirmed by morphological assessment under the microscope
Mycoplasma contamination	The cell lines were regularly tested for mycoplasma contamination, and no mycoplasma contamination was found.
Commonly misidentified lines<br>(See ICLAC register)	No commonly misidentified cell lines were used.

Animals and other organisms

Policy information about studies involving animals; ARRIVE guidelines recommended for reporting animal research

Laboratory animals	Female BALB/C nude mice aged 4-5 weeks were purchased from Guangdong Medicinal Laboratory Animal Center (Guangzhou, China)
Wild animals	The study did not involve wild animals.
Field-collected samples	This study did not involve field-collected samples.
Ethics oversight	All animal experiments were carried out in strict accordance with the regulations of the Animal Ethical and Welfare Committee of Shenzhen University (AEWC-SZU)

Note that full information on the approval of the study protocol must also be provided in the manuscript.

Flow Cytometry

Plots

Confirm that:

☒ The axis labels state the marker and fluorochrome used (e.g. CD4-FITC).

☒ The axis scales are clearly visible. Include numbers along axes only for bottom left plot of group (a 'group' is an analysis of identical markers).

☒ All plots are contour plots with outliers or pseudocolor plots.

☒ A numerical value for number of cells or percentage (with statistics) is provided.

Methodology

Sample preparation	For apoptosis/necrosis analysis: The treated cells were washed with PBS, collected by trypsinization (without EDTA), resuspended in cold PBS. Afterwards, the obtained cells were stained with Annexin V-FITC/PI according to the manufacturer's instruction and analyzed by flow cytometry.
Instrument	BD FACSCanto SORP
Software	Data collection: CellQuest Pro (version 5.1)<br>Data analysis: FlowJo_V10 software (version 10.7.1)
Cell population abundance	No cell sorting was performed.
Gating strategy	Cells were firstly gated to exclude debris by FSC vs. SSC, followed by gating for singlet cells by FSC-H vs. FSC-A. Then, gates were set for Annexin V-FITC/PI-stained cells using samples from PBS-treated controls.

☒ Tick this box to confirm that a figure exemplifying the gating strategy is provided in the Supplementary Information.
